# A Chiral 2D Sheet for Enhancing GLUT1 Function Through the Interplay of Architecture and Recognition

**DOI:** 10.1002/smll.202512285

**Published:** 2026-02-23

**Authors:** Yerim Kim, Dawoon Lee, Kyuri Kim, Young Yong Kim, Bongjun Yeom, Sunihl Ma, Young‐Hoon Kim, Yongju Kim

**Affiliations:** ^1^ KU‐KIST Graduate School of Converging Science and Technology Korea University Seoul Republic of Korea; ^2^ Beamline division Pohang Accelerator Laboratory Pohang University of Science and Technology Pohang Republic of Korea; ^3^ Department of Chemical Engineering Hanyang University Seoul Republic of Korea; ^4^ School of Chemistry and Energy Sungshin Women's University Seoul Republic of Korea; ^5^ Department of Energy Engineering Hanyang University Seoul Republic of Korea; ^6^ Department of Integrative Energy Engineering Korea University Seoul Republic of Korea; ^7^ Chemical and Biological Integrative Research Center Korea Institute of Science and Technology Seoul Republic of Korea

**Keywords:** chiral sheets, GLUT1 modulation, morphology‐dependent function, supramolecules

## Abstract

Controlling cellular function from the extracellular space requires synthetic materials capable of specific and sustained interactions at the cell membrane. This typically demands a synergy between the material's overall architecture and its molecular‐level recognition motifs. Here, we demonstrate this synergistic principle using a glucose‐based amphiphile that can be assembled into two distinct architectures. While the amphiphile alone forms 0D nanoparticles that are readily internalized by cells, its co‐assembly with a molecular trigger yields 2D nanosheets that remain on the cell exterior. This 2D morphology provides the necessary platform for sustained cell‐surface interaction, while the chirality of the glucose units acts as the specific recognition key. We show that only the 2D sheets presenting d‐glucose effectively upregulate the membrane protein GLUT1 and trigger a downstream antioxidant response. This function is absent for the internalized 0D particles and the enantiomeric (l)‐sheets, providing a clear demonstration of the powerful and essential synergy between supramolecular morphology and molecular chirality for the precise control of cellular behavior.

## Graphical abstract

We show that both supramolecular architecture and glucose chirality determine biological function. Unlike self‐assembled 0D particles, we assembled with OFN to form 2D sheets. Only the (d)‐sheets with exposed d‐glucose interacted with GLUT1, increasing its expression, promoting glucose uptake, and reducing ROS. This highlights a synergistic design principle of chiral, cell‐impermeable surfaces to inspire therapeutic biomaterials.

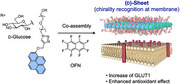

## 1 Introduction

A central goal in materials chemistry is to design synthetic materials that can precisely control complex biological processes [1, 2, 3, 4]. A key arena for such control is the cell membrane, the primary interface for cellular communication [5, 6, 7, 8, 9, 10]. While 0D nanoparticles are widely used as delivery vehicles, their rapid internalization often prevents them from acting as functional platforms for sustained, specific interactions at the cell surface [11, 12, 13, 14, 15, 16]. To create materials that can actively modulate membrane‐level events, a more sophisticated design strategy is required, one that moves beyond simple passive structures.

We propose that the precise control of membrane protein function can be achieved through a powerful synergy between a material's macroscopic architecture and its molecular‐level recognition elements. Specifically, a 2D morphology can provide a platform that, unlike 0D particles, resists internalization and offers a large surface area for cell interaction [17, 18, 19, 20, 21, 22, 23]. Concurrently, molecular chirality can be encoded onto this platform to serve as a highly specific key for recognizing and engaging target proteins within the inherently chiral environment of the cell membrane [24, 25, 26, 27, 28]. The combination of the right platform (2D structure) and the right key (correct chirality) should therefore unlock functions that neither component can achieve alone.

**FIGURE 1:**
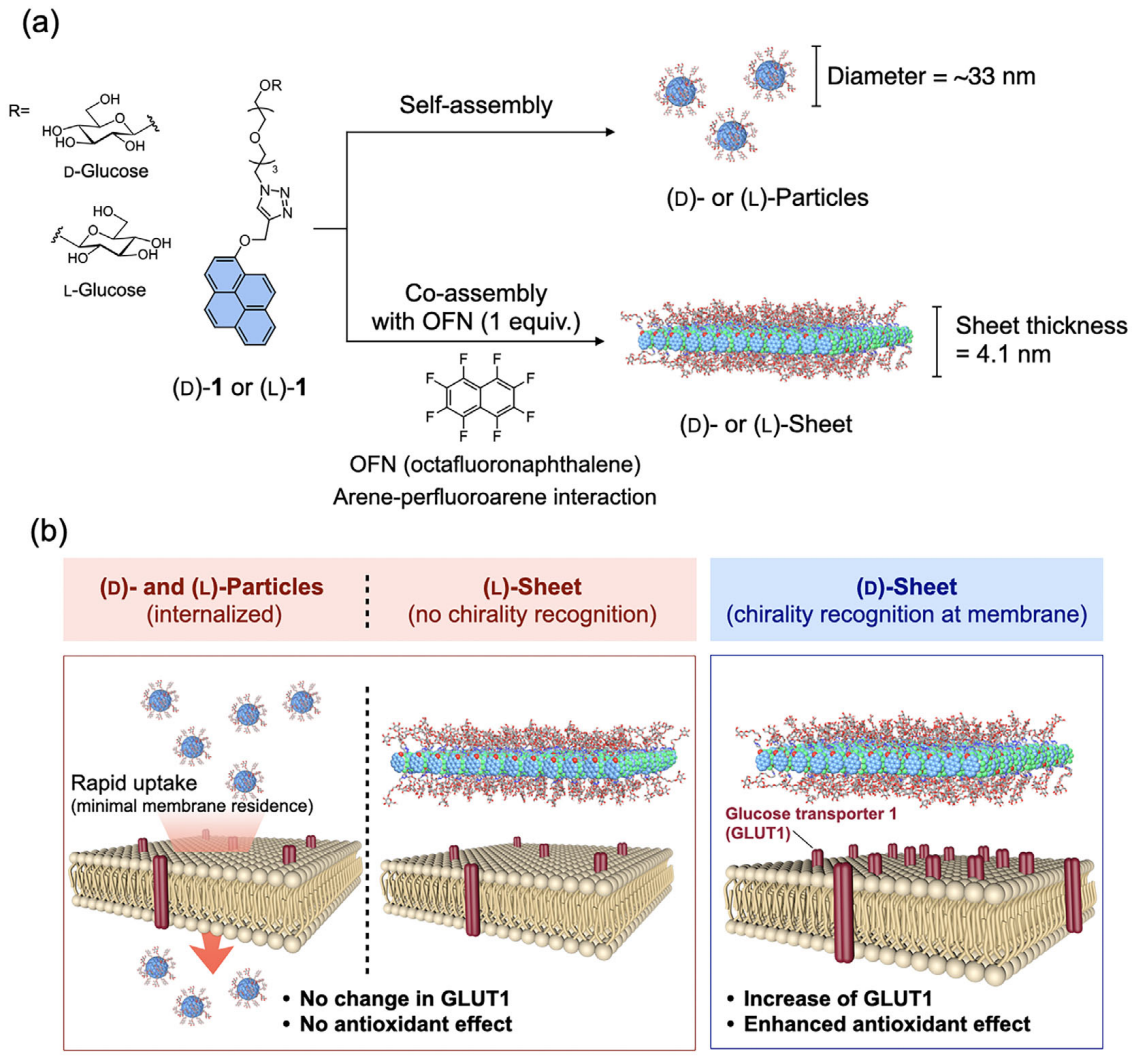
Schematic illustration of the formation of supramolecular nanoparticles and chiral sheets and their distinct biological functions. (a) Glucose‐based chiral amphiphiles, (d)‐**1** or (l)‐**1**, self‐assembles into 0D nanoparticles. Co‐assembly with octafluoronaphthalene (OFN) triggers a morphological transformation into 2D sheets. (b) Summary of cellular responses. Only the (d)‐sheet, which remains on the cell exterior, specifically upregulates glucose transporter 1 (GLUT1), leading to an enhanced antioxidant effect.

Herein, we provide a powerful demonstration of this synergistic principle. Based on previously studied aromatic‐glycoside conjugates, we designed and synthesized a glucose‐based chiral amphiphile [29, 30]. We found that this molecule can form either 0D nanoparticles or grow into large 2D nanosheets through co‐assembly with a trigger molecule, depending on the assembly conditions (Figure 1a). We demonstrate that these two architectures have fundamentally different biological fates and functions. While the particles are internalized, the sheets remain on the cell surface, where a powerful synergy between the 2D morphology and the d‐glucose chirality enables the specific upregulation of the glucose transporter 1 (GLUT1) and a subsequent intracellular antioxidant response (Figure 1b). This study establishes a clear design strategy where the synergy between supramolecular structure and molecular chirality can be harnessed to control cellular machinery from the outside‐in.

## 2 Results and Discussion

**SCHEME 1:**
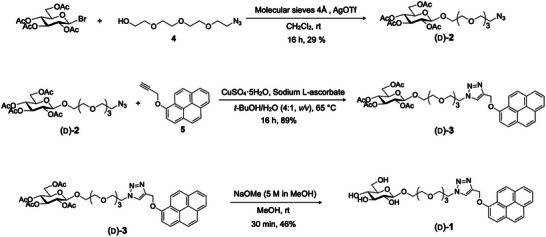
Synthetic route to glucose‐based chiral amphiphiles (d)‐**1**.

The chiral amphiphiles (d)‐**1** and (l)‐**1** were synthesized via a four‐step sequence starting from commercially available d‐ and l‐glucose, respectively (Scheme 1). Key strategic steps included the preparation of the acetylated glycosyl bromide donor, installation of a hydrophilic linker via glycosylation with 2‐(2‐(2‐azidoethoxy) ethoxy) ethanol (molecule 4), and attachment of the hydrophobic pyrene unit using a copper‐catalyzed ‘click’ reaction with 1‐propargyloxy pyrene (molecule 5). During the glycosylation step, the relatively low yield obtained with the peracetylated glycosyl donor was attributed to competing side reactions commonly associated with acetobromo sugars, such as glycosyl orthoester formation. To assess the influence of donor protecting groups, the acetyl groups were replaced with benzoyl groups, which led to an improved isolated yield from 29% to 42%. In the final step, global deprotection of the acetyl groups furnished the target amphiphiles (d)‐**1** and (l)‐**1**. The enantiomeric nature of the final products was further confirmed by specific optical rotation measurements at 589 nm, which showed comparable magnitudes with opposite signs: (d)‐**1**, [α]_D_^25^ = −6.7 (c = 6.5 × 10^−3^ g mL^−1^, MeOH) and (l)‐**1**, [α]_D_^25^ = +6.9 (c = 6.5 × 10^−3^ g mL^−1^, MeOH). The structures of the final products and all intermediates were rigorously characterized by nuclear magnetic resonance (NMR) spectroscopy and mass spectrometry (see Supporting Information, Figures S1−S14).

**FIGURE 2:**
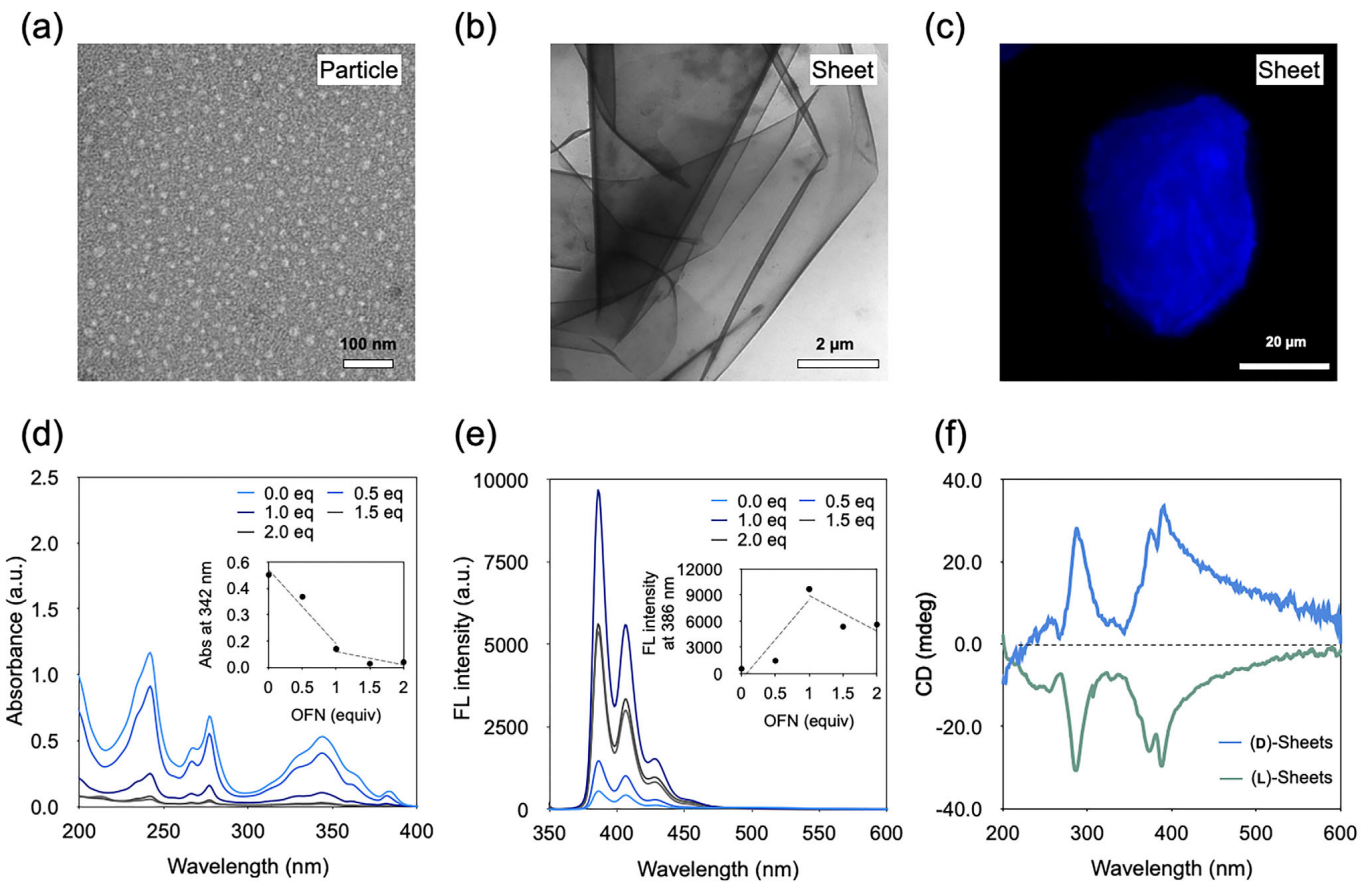
Morphological and spectroscopic characterization of the OFN‐triggered transition from particles to 2D sheets at 314 µM. (a,b) Transmission electron microscopy (TEM) images showing the morphological change from (d)‐**1** nanoparticles to large 2D sheets after adding OFN. (c) A micrometer‐scale sheet observed by fluorescence optical microscopy (FOM). (d, e) UV–vis absorption and fluorescence emission spectra indicating strong intermolecular π‐stacking upon OFN addition. (f) Circular dichroism (CD) spectra confirming the transfer of molecular chirality to the supramolecular assembly.

We first investigated the self‐assembly behavior of our synthesized chiral amphiphile, (d)‐**1**, in an aqueous solution. Transmission electron microscopy (TEM) revealed that (d)‐**1** formed uniform, spherical particles (Figure 2a and Figure S15), and dynamic light scattering (DLS) analysis confirmed their average diameter to be approximately 33 nm (Figures S16 and S17). To probe the structural diversity accessible from these nanoparticle precursors, we introduced octafluoronaphthalene (OFN) to the solution. Based on the known arene‐perfluoroarene (AP) π‐stacking interaction, we investigated whether the π‐stacking interaction between electron‐rich pyrene and electron‐deficient OFN of (d)‐**1** induces morphological transformation [18, 19, 31].

The co‐assembly process was examined by first dissolving (d)‐**1** and OFN completely in methanol to afford a homogeneous solution. The solvent was then removed by evaporation to form a dry film, which was subsequently rehydrated with water, thereby inducing co‐assembly under aqueous conditions. This prompted distinct spectral changes; the pyrene absorption band at 342 nm exhibited a pronounced hypochromic shift (Figure 2d and Figure S18), while the fluorescence emission intensity at 386 nm was significantly enhanced (Figure 2e and Figure S19). Simultaneously, the circular dichroism (CD) ellipticity also increased (Figure S20). These spectral changes are indicative of strong intermolecular π‐stacking between the pyrene moiety of (d)‐**1** and OFN. All spectra saturated at 1.0 equivalent of OFN, establishing this as the optimal stoichiometry. Crucially, the transfer of molecular‐level chirality to the ensemble was confirmed by CD spectroscopy. The (d)‐ and (l)‐sheets displayed intense and perfectly mirror‐imaged CD spectra, confirming the formation of large‐scale chiral assemblies (Figure 2f and Figures S21–S24). This spectroscopically observed transformation was then directly visualized. TEM images revealed a dramatic morphological shift from the discrete nanoparticles to extensive, well‐defined 2D sheets several micrometers in size (Figure 2b and Figures S25−S27). The large, micrometer‐scale dimensions of these sheets were further corroborated in solution by fluorescence optical microscopy (FOM), optical microscopy (OM), and DLS (Figure 2c and Figures S16, and S28). These comprehensive results confirm that the addition of OFN serves as a robust trigger for the co‐assembly of (d)‐**1** particles into large, stable, and chiral 2D nanosheets.

**FIGURE 3:**
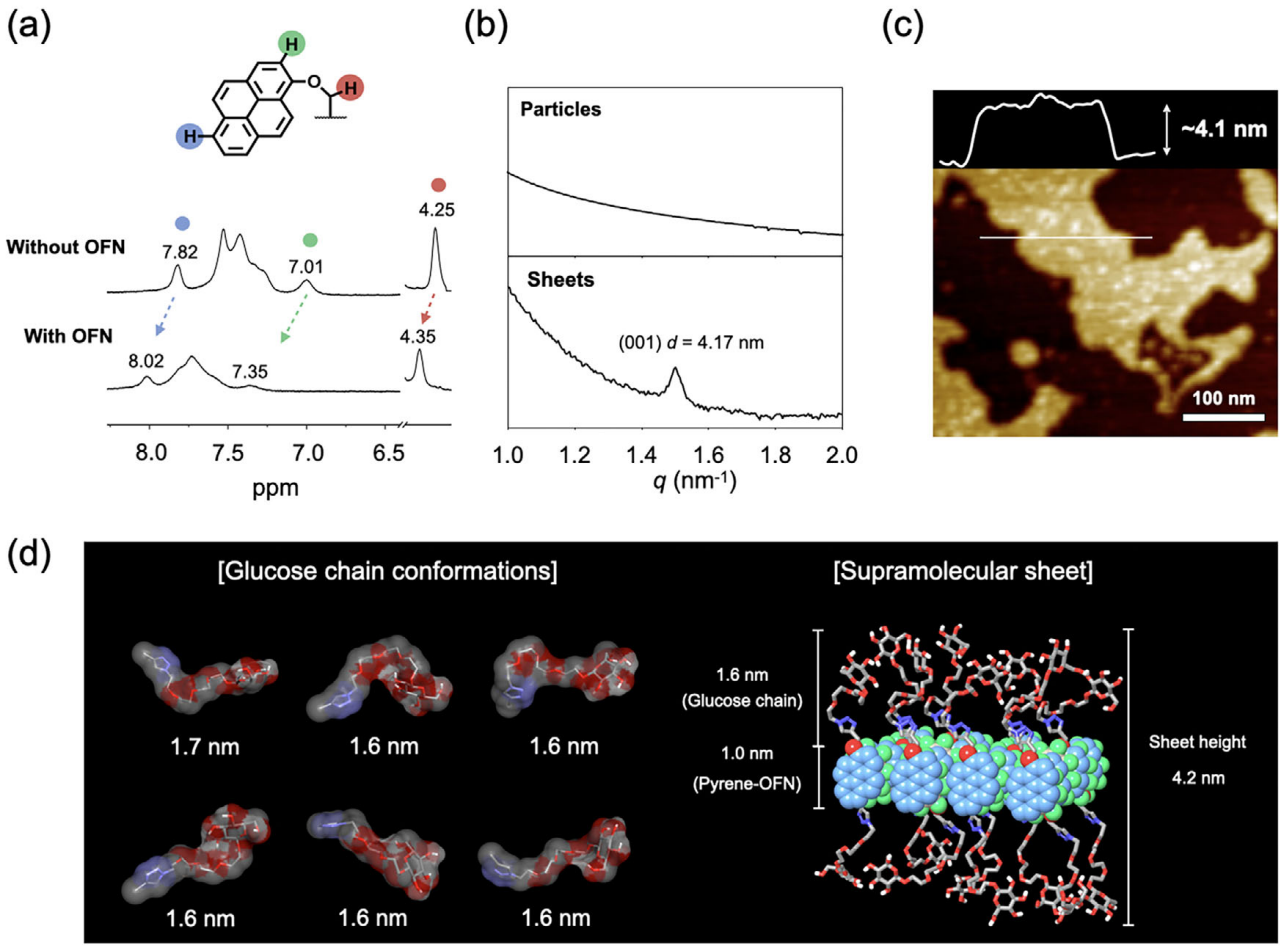
Detailed structural analysis of the 2D sheets. (a) ^1^H NMR spectra showing downfield shifts of pyrene protons, consistent with arene‐perfluoroarene π‐stacking. (b) Small‐angle X‐ray scattering (SAXS) profiles revealing a distinct *d*‐spacing of 4.17 nm for the sheets. (c) Atomic force microscopy (AFM) height image confirming a uniform sheet thickness of ∼4.1 nm. (d) A molecular model from molecular dynamics (MD) simulations consistent with experimental results.

To elucidate the detailed molecular arrangement within the 2D nanosheets, we conducted a series of structural analyses. First, ^1^H NMR spectroscopy in deuterium oxide (D_2_O) provided direct evidence of the AP interaction at the molecular level. Upon sheet formation, the aromatic proton signals of the pyrene moiety exhibited a noticeable downfield shift compared to those of the precursor particles, confirming a decrease in electron density consistent with π‐π stacking between the pyrene moiety of (d)‐**1** and the electron‐deficient OFN (Figure 3a).

The sheet structure of the assemblies was then characterized by X‐ray scattering and microscopy. Small‐angle X‐ray scattering (SAXS) analysis showed a distinct diffraction peak corresponding to a *d*‐spacing of ∼4.17 nm for the sheets, a feature absents in the unstructured particle sample (Figure 3b). This uniform sheet thickness was independently corroborated by atomic force microscopy (AFM), which revealed a consistent height of approximately 4.1 nm (Figure 3c). To probe the internal order, wide‐angle X‐ray scattering (WAXS) was performed. The sheets displayed a strong reflection at *q* corresponding to a distance of ∼3.4 Å, the characteristic spacing for highly ordered, face‐to‐face π‐π stacking, which was absent in the amorphous particle sample (Figure S29).

To integrate these experimental findings into a coherent molecular model, we performed molecular dynamics (MD) simulations. The simulations indicated that the hydrophilic segment, consisting of the glucose and triethylene glycol chain, has an average extended length of ∼1.6 nm. When combined with the estimated ∼1.0 nm thickness of the pyrene‐OFN π‐stacked core, the total calculated sheet thickness is ∼4.2 nm (Figure 3d and Figure S30). This computationally derived value is in excellent agreement with the experimental data from both SAXS and AFM. Taken together, these multi‐scale analyses provide a comprehensive and consistent structural model. The results show the nanosheets are highly ordered, crystalline monolayers formed by the specific co‐assembly of (d)‐**1** and OFN. This assembly is driven by π‐π interactions and results in a uniform thickness dictated by the length of the hydrophilic glucose‐bearing chains.

**FIGURE 4:**
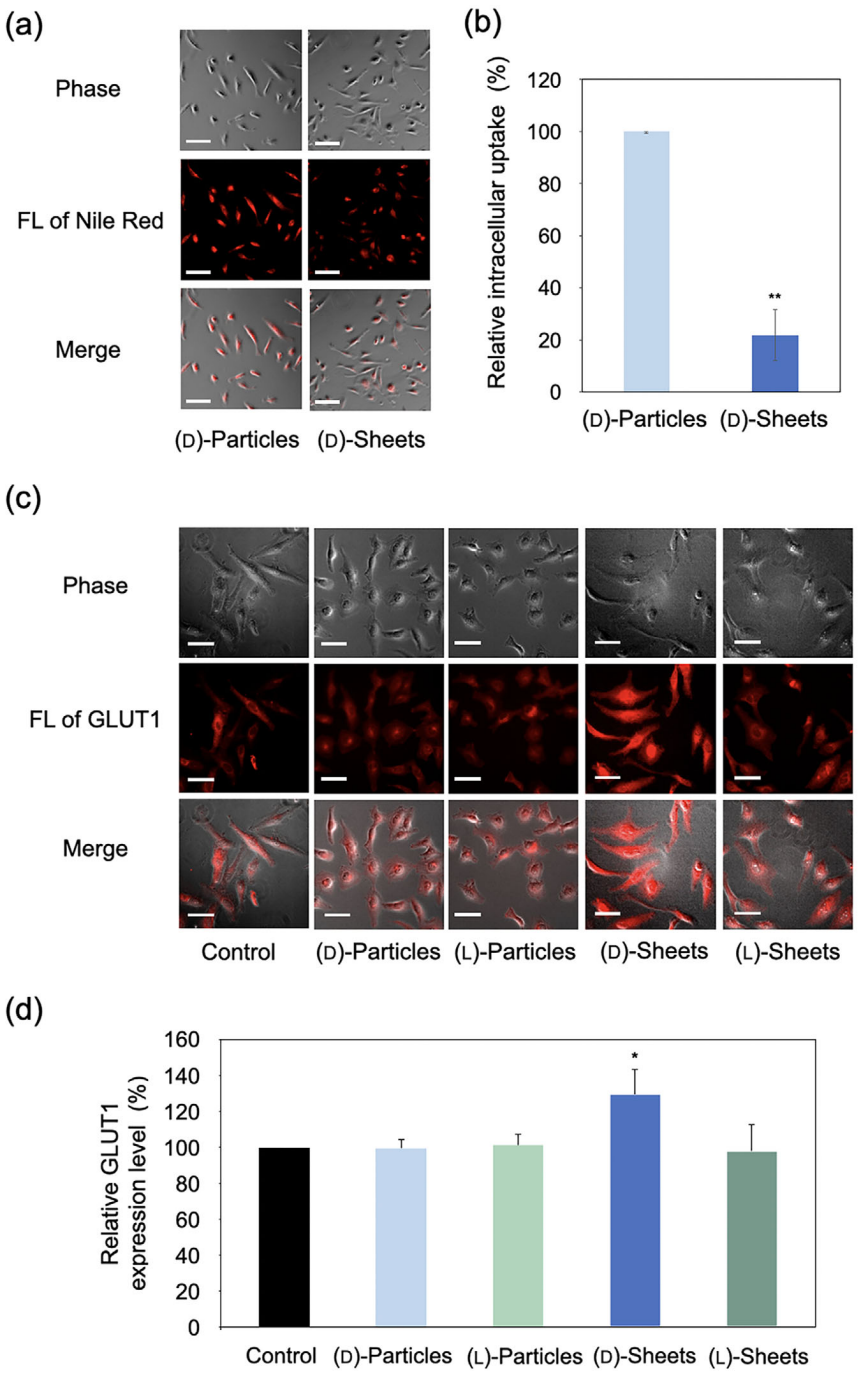
Differential cellular uptake and specific GLUT1 upregulation by chiral sheets at 50 µm. (a, b) Fluorescence optical microscopy (FOM) and quantification show that the large 2D sheets exhibit ∼80% less cellular uptake compared to the nanoparticles. (c, d) Immunofluorescence images and quantification reveal a significant (∼30%) increase in GLUT1 expression only in cells treated with (d)‐sheets. The data are represented as mean ± SD (*n* = 3). ^*^ *p* < 0.05, ^**^ *p* < 0.001.

Having established the distinct supramolecular structures, we next investigated how their different morphologies and chiralities dictate their interactions with living cells. A key objective of our design was that the 2D sheet architecture, in contrast to 0D particles, would resist cellular internalization and thus be positioned for sustained interactions at the cell membrane interface. To verify this, we first compared the cellular uptake of (d)‐particles and (d)‐sheets in HeLa cells using Nile Red as a fluorescent probe. FOM images revealed a clear difference. The compact, ∼33 nm (d)‐particles were readily internalized by the cells, resulting in strong intracellular fluorescence (Figure 4a). In contrast, the micrometer‐scale (d)‐sheets were largely excluded from the cell interior and did not produce a significant intracellular signal. This visual observation was quantified, confirming an ∼80% reduction in intracellular accumulation for the sheets compared to the particles (Figure 4b). This result is critical, as it confirms that the large lateral dimensions of the sheets effectively prevent cellular uptake, validating their design as a platform for specific cell‐surface recognition rather than intracellular delivery.

This effective exclusion from the cell interior prompted us to investigate whether the sheets could modulate membrane protein function at the cell surface. We focused on GLUT1, a glucose transporter whose activity is sensitive to the stereochemical context of d‐glucose [32]. Notably, the observed cellular response did not arise from free glucose units, but emerged only when d‐glucose was presented within a membrane‐resident 2D supramolecular architecture. HeLa cells were therefore treated with all four supramolecular structures and analyzed by immunocytochemistry (ICC) to visualize GLUT1 expression. Strikingly, only cells treated with the (d)‐sheets exhibited a pronounced upregulation of GLUT1, appearing as a dense network of fluorescent signals (Figure 4c). Quantitative analysis confirmed an approximately 30% increase in GLUT1 levels for the (d)‐sheets, while neither the internalized (d)‐particles nor the enantiomeric (l)‐sheets and (l)‐particles produced any significant change (Figure 4d). These results indicate that both a membrane‐resident 2D architecture and the correct stereochemical presentation of d‐glucose are required to elicit the observed GLUT1‐associated cellular response in our system.

Finally, we performed experiments to confirm that this observed upregulation in protein expression translated into a direct functional consequence. Using the fluorescent glucose analog 2‐deoxy‐2‐[(7‐nitro‐2,1,3‐benzoxadiazol‐4‐yl)amino]‐D‐glucose (2‐NBDG), we measured the rate of glucose transport into the cells. Consistent with the ICC data, cells treated with the (d)‐sheets demonstrated a significantly enhanced glucose uptake—approximately 35% higher than cells treated with the inactive (l)‐sheets (Figures S31 and S32). Importantly, control experiments below the critical aggregation concentration show that monomeric (d)‐**1** does not affect glucose uptake, indicating that the biological response is not triggered by individual glucose units. Instead, the response emerges only when glucose moieties are organized within a stable, membrane‐associated 2D architecture. To definitively prove that this enhanced transport was mediated by the upregulated GLUT1, we repeated the experiment in the presence of cytochalasin B, a known GLUT1 inhibitor [33, 34]. This inhibitor reduced cellular uptake by approximately 30% in (d)‐sheet‐treated cells compared to the control group (Figure S33). Taken together, these data support a consistent model in which the (d)‐sheets, by virtue of their morphology and chirality, remain on the cell surface, where their 2D, chiral presentation induces a cellular response involving GLUT1 upregulation and enhanced glucose transport.

Based on the finding that the (d)‐sheets upregulates functional GLUT1 expression, we next sought to determine the subsequent functional outcomes of this interaction. A direct link between GLUT1‐mediated glucose transport and the regulation of intracellular reactive oxygen species (ROS) has been previously established, with studies showing that increased glucose uptake can enhance cellular antioxidant capacity [35]. We therefore investigated whether the (d)‐sheets‐induced upregulation of GLUT1 would translate to a protective effect against oxidative stress. To this end, we induced oxidative stress in HeLa cells using *tert*‐butyl hydroperoxide (TBHP) and then measured ROS levels using a 2’,7’‐dichlorodihydrofluorescein diacetate (DCFDA) assay.

**FIGURE 5:**
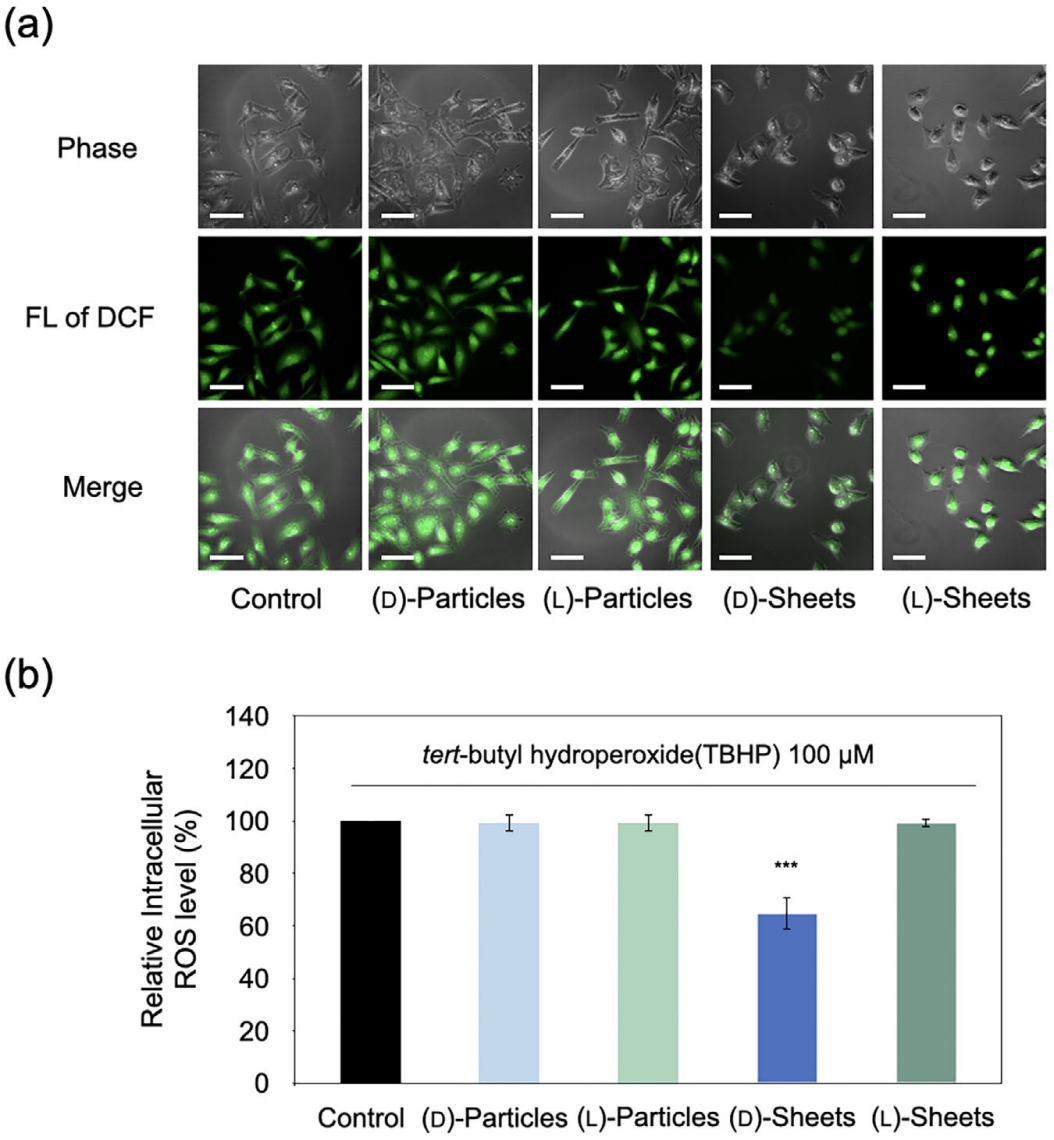
The (d)‐sheets confer a potent antioxidant effect by reducing intracellular reactive oxygen species (ROS) at 50 µM. (a) Fluorescence images of DCF, indicating intracellular ROS levels in *tert*‐butyl hydroperoxide (TBHP)‐stressed HeLa cells. (b) Quantification shows that treatment with (d)‐sheets resulted in a ∼40% reduction in ROS, while other control groups showed no significant antioxidant activity. The data are represented as mean ± SD (*n* = 3). ^***^ *p* < 0.001.

FOM images revealed a significant reduction in ROS‐associated fluorescence exclusively in cells treated with the (d)‐sheets (Figure 5a). This antioxidant effect was confirmed by quantitative analysis, which showed an approximate 40% decrease in ROS levels. In contrast, no significant antioxidant activity was observed for the internalized (d)‐particles, the (l)‐particles, or the enantiomeric (l)‐sheets, all of which failed to reduce ROS levels (Figure 5b). This result provides strong evidence that the upregulation of GLUT1 is a key factor in enhancing the cell's intrinsic antioxidant capacity, consistent with previous reports.

Crucially, the lack of activity from the (l)‐sheets again highlights the importance of molecular chirality in this biological recognition process. This indicates that it is not merely the 2D sheet structure interacting with the membrane, but the specific, chirality‐dependent involvement of GLUT1‐associated pathways associated with the d‐glucose‐presenting sheet surface that is responsible for the observed biological effects, from protein expression to functional antioxidant activity. Therefore, our results demonstrate that a synthetic supramolecular sheet bearing the correct chirality can lead to upregulation of functional GLUT1 expression and, as a functional consequence, enhance the cell's intrinsic antioxidant defenses. Taken together, these results indicate that the enhanced glucose uptake does not originate from direct substrate recognition, but from a morphology‐ and chirality‐dependent cellular response elicited by the membrane‐resident 2D sheets.

## 3 Conclusion

In summary, we have demonstrated that both the supramolecular architecture (0D particle vs. 2D sheet) and the molecular‐level chirality of a glucose‐based amphiphile are critical determinants of its biological function. While the self‐assembled nanoparticles were readily internalized by cells, the co‐assembly of the amphiphile with OFN yielded micrometer‐sized 2D sheets that were excluded from the cell interior, positioning them for sustained interactions at the cell membrane. This distinct membrane localization enabled a remarkable functional outcome. We found that the (d)‐sheets, presenting d‐glucose on their surface, selectively induce a GLUT1‐mediated increase in glucose transport. This interaction triggers a significant upregulation of functional GLUT1 expression, leading to enhanced glucose uptake and, consequently, a potent intracellular antioxidant effect evidenced by a ∼40% reduction in ROS levels. Crucially, the absence of this effect in the enantiomeric (l)‐sheets and the internalized (d)‐particles demonstrates that the observed antioxidant response arises from a synergistic effect between the 2D sheet morphology, and the correct d‐glucose chirality presented on its surface.

This work establishes a powerful design principle involving the use of synthetic, cell‐impermeable, chiral surfaces to control a specific intracellular metabolic pathway from the outside‐in. This strategy of harnessing specific molecular recognition at the cell membrane opens new avenues for the development of sophisticated biomaterials capable of precisely modulating cellular behavior for applications in metabolic engineering, cell signaling, and targeted therapeutics.

## 4 Experimental Section

### 4.1 General Methods

Unless otherwise noted, all materials, organic reagents, and solvents were purchased from commercial suppliers (TCI, Aldrich, Aladdin, etc.). Anhydrous dichloromethane (DCM) was distilled from CaH_2_, and deionized water (DW) was obtained through ion exchange and filtration. Thin‐layer chromatography (TLC) was performed on silica gel 60 F254 glass plates (0.25 mm thickness), and components were visualized under UV light (254 and 365 nm) or by staining with ceric ammonium molybdate (CAM) or KMnO_4_ solutions. Products were purified using flash column chromatography on silica gel (230–400 mesh). Mass spectra were collected on an Advion Expression CMS mass spectrometer. Analytical and preparative high‐performance liquid chromatography (HPLC) were carried out on a Shimadzu LC‐ 20AR equipped with YMC‐Pack Pro C18 (analytical, 250 × 4.6 mm i.d.) and YMC‐Actus Triart C18 (preparative, 250 × 20.0 mm i.d.) columns, respectively. ^1^H and ^13^C nuclear magnetic resonance (NMR) spectra were recorded on a 400 MHz FT‐NMR spectrometer (JNM‐ECZ400S/L1). UV–visible absorption spectra were obtained using an Agilent 8453E UV‐visible spectrophotometer, and fluorescence emission spectra were measured with a Hitachi F‐7000 fluorescence spectrophotometer. Circular dichroism (CD) spectra were acquired with a JASCO J‐1100 spectropolarimeter. The specific optical rotation measurements were performed using a JASCO P‐2000 spectropolarimeter at 589 nm and 25°C with a 10 mm path length cell. Dynamic light scattering (DLS) measurements were performed at a fixed angle of 90° using an Otsuka Electronics ELSZ‐1000 to determine the size distribution of supramolecular structures. Transmission‐mode X‐ray scattering experiments were carried out at the Pohang Accelerator Laboratory using synchrotron radiation.

### 4.2 Sampling Methods

(d)‐**1** and OFN were each dissolved in MeOH and mixed to obtain the desired concentration. The solvent was removed under reduced pressure, and DW was added to the dried residue to prepare a solution with the desired final concentration. The resulting aqueous mixture was sonicated in a cold‐water bath for 60 min.

### 4.3 Transmission Electron Microscopy (TEM) Experiments

A 2 µL aliquot of the sample solution was applied onto a carbon‐coated grid (Carbon Type B, 200 mesh with Formvar; Ted Pella, Inc.). After drying under ambient conditions, the grid was stained with an aqueous solution of uranyl acetate (0.4 wt.%). The specimens were examined using a Hitachi H‐7650 transmission electron microscope operated at 100 kV.

### 4.4 Nuclear Magnetic Resonance (NMR) Experiments

^1^H NMR spectra (400 MHz) were obtained for (d)‐**1** with and without OFN in D_2_O/MeOD (9:1, *v/v*) at a concentration of 314 µm.

### 4.5 Atomic Force Microscope (AFM) Experiments

A 2 µL of the sample solution was deposited onto mica and allowed to dry under ambient conditions. Atomic force microscopy (AFM) measurements were performed using an NX‐10 instrument (Park Systems) at room temperature. XEI software (Park Systems) was used for image analysis.

### 4.6 Molecular Simulations

The initial structures were subjected to energy minimization using the MacroModel module of Schrödinger Suites (Schrödinger K.K.) using the OPLS4 force field. Water was used as the solvent (cutoff: none; minimization method: PRCG; maximum iterations: 2500; converge on gradient; convergence threshold: 0.05). Molecular dynamics (MD) simulations were performed via the Desmond module in Schrödinger Suites (Schrödinger K.K.) with the following parameters‐force field: OPLS4; solvent model: SPC; boundary conditions: orthorhombic box shape; simulation time: 20 ns; ensemble class: NPT; temperature: 300 K; pressure: 1.01325 bar; thermostat method: Nose– Hoover chain; coulombic interaction cutoff radius: 9.0 Å.

### 4.7 Cell culture

HeLa cells (Korean Cell Line Bank, Seoul, Korea) were cultured in Dulbecco's modified Eagle's medium (DMEM; HyClone, Logan, UT, USA), 10% (*v/v*) fetal bovine serum (FBS; Gibco; Thermo Fisher Scientific), and 1% penicillin/streptomycin (P/S: HyClone, Logan, UT, USA). The culture medium was replaced with fresh medium when the cells reached 90–95% confluence. The HeLa cells were cultured at 37°C with an atmosphere of 5% CO_2_ in a humidified incubator.

### 4.8 Fluorescence Optical Microscopy (FOM) Experiments

HeLa cells were seeded in 13‐mm confocal dishes at a density of 20,000 cells per well and cultured for 24 h. The cells were subsequently treated for 2–4 h, washed with phosphate‐buffered saline (PBS), and imaged using FOM (Leica DMi8). Image acquisition and analysis were performed with LAS X software (Leica Microsystems).

### 4.9 Immunocytochemistry

HeLa cells were seeded in black 96‐well plates (SPL, Korea) at a density of 20,000 cells per well and incubated for 24 h. The cells were washed with phosphate‐buffered saline (PBS), fixed with 4% formaldehyde (Sigma–Aldrich) for 10 min, and permeabilized with 0.2% Triton X‐100 (Sigma–Aldrich) for 5 min. After washing with PBS, the cells were blocked for 1 h with blocking buffer containing 1% bovine serum albumin (BSA), 10% normal goat serum, and 0.3 M glycine in 0.1% PBS‐Tween. Subsequently, the cells were incubated overnight at 4°C in the dark with Alexa Fluor 594–conjugated anti‐GLUT1 antibody (1:100 dilution, Abcam, ab206360) prepared in PBS. Following antibody incubation, the cells were washed with PBS, and fluorescence intensity was measured using a Cytation 3 reader (BioTek, USA) at λ_ex_ = 590 nm and λ_em_ = 617 nm.

### 4.10 2‐NBDG Assay

HeLa cells were seeded at a density of 20,000 cells per well in black 96‐well plates (SPL, Korea) and cultured overnight. The cells were incubated in glucose‐free medium for and subsequently treated with the fluorescent glucose analog 2‐deoxy‐2‐[(7‐nitro‐2,1,3‐ benzoxadiazol‐4‐yl)amino]‐D‐glucose (2‐NBDG) (100–200 µg/mL) in glucose‐free medium for 15 min. Following a final PBS wash, the cells were resuspended in PBS, and fluorescence intensity was measured using a Cytation 3 reader (BioTek, USA) at λ_ex_ = 465 nm and λ_em_ = 540 nm.

### 4.11 DCFDA Assay

HeLa cells were seeded at a density of 20,000 cells per well in black 96‐well plates (SPL, Korea) and cultured overnight. The cells were incubated in phenol red–free DMEM and treated with *tert*‐butyl hydroperoxide (TBHP, 100 µM) and individual samples (50 µM) for 2 h. After treatment, the cells were washed with PBS and incubated with DCFDA (20 µM) for 45 min. Following a final PBS wash, the cells were resuspended in PBS, and fluorescence intensity was measured using a Cytation 3 reader (BioTek, USA) at λ_ex_ = 485 nm and λ_em_ = 535 nm.

### 4.12 GLUT1 Inhibitor Assay

HeLa cells were seeded in black 96‐well plates (SPL, Korea) at a density of 20,000 cells per well and incubated for 24 h. The cells were then washed with phosphate‐buffered saline (PBS) and pre‐treated with 1.3 µM cytochalasin B for 30 min. After PBS washing, the cells were treated with particles and sheets (50 µm) for 2 h. Following treatment, the cells were washed with PBS, and the fluorescence intensity was measured using a Cytation 3 imaging reader (BioTek, USA) at λ_ex_ = 342 nm and λ_em_ = 386 nm.

### 4.13 Statistical Analysis

Raw data were normalized prior to statistical analysis. All experiments were carried out in triplicate, and the data are expressed as mean values ± standard deviation (SD). For statistical analyses, Student's *t*‐test and one‐way ANOVA followed by Tukey's multiple comparison test were performed to compare differences among groups. A *p*‐value < 0.05 was considered statistically significant (GraphPad Prism, San Diego, CA).

## Conflicts of Interest

The authors declare no conflicts of interest.

## Supporting information




**Supporting File**: smll72897‐sup‐0001‐SuppMat.pdf.

## Data Availability

The data that support the findings of this study are available from the corresponding author upon reasonable request.
