# Supplementary material for: A Chiral 2D Sheet for Enhancing GLUT1 Function Through the Interplay of Architecture and Recognition
Source: Small. 2026 Feb 23;22(23):e12285. doi: 10.1002/smll.202512285 (PMC13100554; doi:10.1002/smll.202512285)
Supplement: Supplementary file 1 — Supporting File: smll72897‐sup‐0001‐SuppMat.pdf. [file SMLL-22-e12285-s001.pdf]

Supporting Information

A Chiral 2D Sheet for Controlling GLUT1 Function through the Interplay of Architecture and Recognition

Yerim Kim[a], Dawoon Lee[a], Kyuri Kim[a], Young Yong Kim[b], Bongjun Yeom[c], Sunihl Ma[d], Young-Hoon Kim[e], Yongju Kim[a], [f], [g]*

[a] Y. Kim, D. Lee, K. Kim, Prof. Y. Kim
KU-KIST Graduate School of Converging Science and Technology
Korea University, Seoul 02841, Republic of Korea
E-mail: yongjukim@korea.ac.kr
[b] Y. Y. Kim
Beamline division, Pohang Accelerator Laboratory
Pohang University of Science and Technology, Pohang 37673, Republic of Korea
[c] Prof. B. Yeom
Department of Chemical Engineering
Hanyang University, Seoul 24763, Republic of Korea
[d] Prof. S. Ma
School of Chemistry and Energy
Sungshin Women's University, Seoul 01133, Republic of Korea
[e] Prof. Y.-H. Kim
Department of Energy Engineering
Hanyang University, Seoul 04763, Republic of Korea
[f] Prof. Y. Kim
Department of Integrative Energy Engineering
Korea University, Seoul 02841, Republic of Korea
[g] Prof. Y. Kim
Chemical and Biological Integrative Research Center
Korea Institute of Science and Technology, Seoul 02792, Republic of Korea

Section 1. Synthetic methods

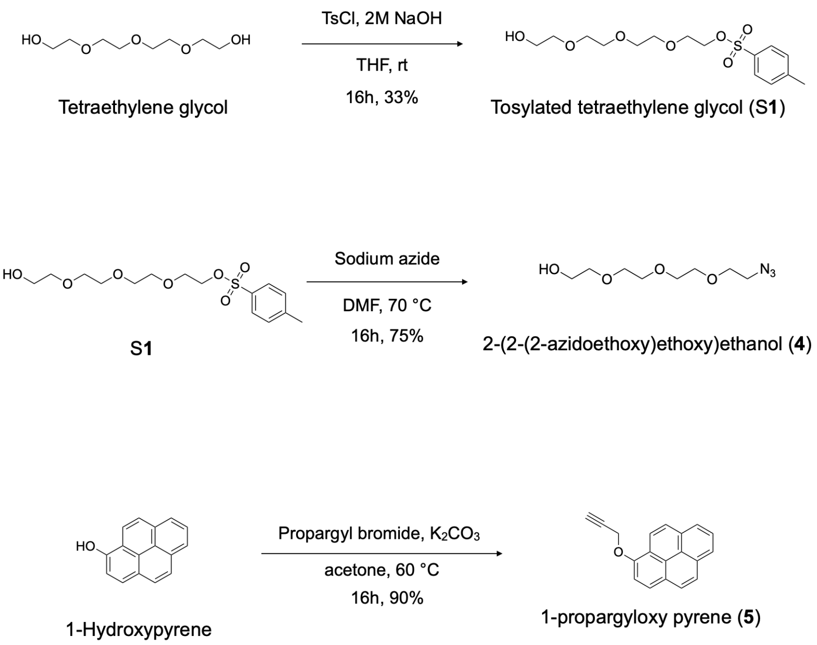

Scheme S1. Synthetic methods of molecule S**1**, molecule **4**, and molecule **5**.

Synthesis of molecule S1

Tetraethylene glycol (1 g, 5.15 mmol) was dissolved in tetrahydrofuran (THF), and 3 mL of 2 M sodium hydroxide (NaOH) was added. A solution of *p*-toluenesulfonyl chloride (TsCl) (687 mg, 3.60 mmol) in THF was added dropwise to the reaction mixture, which was stirred at room temperature for 16 h. The reaction progress was monitored by TLC. The mixture was extracted with dichloromethane (DCM), and the organic phase was dried over anhydrous magnesium sulfate ($MgSO_4$). The filtrate was concentrated under reduced pressure, and the crude product was purified by silica gel flash column chromatography (ethyl acetate: n-hexane 2:1, *v/v*) to provide a light-yellow oil (594.4 mg, 33% yield).

^{1}H NMR (400 MHz, $CDCl_3$): δ 7.79 (d, *J* = 8.4 Hz, 2H), 7.33 (d, *J* = 8.0 Hz, 2H), 4.17–4.15 (m, 2H), 3.72–3.58 (m, 14H), 2.44 (s, 3H). ESI mass: *m/z* calcd for $C_{15}H_{24}O_7S$ $[M+Na]^+$, 371.1; found: 371.0.

Synthesis of molecule 4

Molecule S**1** (300 mg, 0.86 mmol) was dissolved in dimethylformamide (DMF), and sodium azide (312.5 mg, 4.8 mmol) in DMF was added dropwise. The mixture was stirred at 70 °C for 16 h, and the reaction progress was monitored by TLC using ceric ammonium molybdate (CAM) staining. The reaction mixture was extracted with dichloromethane, and the organic phase was dried over anhydrous $MgSO_4$. After concentration under reduced pressure and drying under vacuum, the product was obtained as a yellow oil (185.5 mg, 98% yield).

^{1}H NMR (400 MHz, $CDCl_3$): δ 3.73 (dd, *J* = 6.1, 3.2 Hz, 2H), 3.69–3.68 (m, 10H), 3.69–3.60 (m, 2H), 3.41–3.38 (m, 2H). ESI mass: *m/z* calcd for $C_8H_{17}N_3O_4$ $[M+Na]^+$, 242.1, $[M+K]^+$, 257.1; found: $[M+Na]^+$, 242.6, $[M+K]^+$, 257.9.

Synthesis of molecule 5

1-Hydroxypyrene (300 mg, 1.37 mmol) was dissolved in acetone, and a solution of propargyl bromide (490 mg, 4.12 mmol) and potassium carbonate (500 mg, 2.7 mmol) in acetone was added dropwise. The mixture was stirred at 60 °C for 16 h, and the reaction progress was monitored by TLC. The crude product was extracted with dichloromethane, and the organic phase was dried over anhydrous $MgSO_4$. Solvent removal under reduced pressure followed by vacuum drying afforded the product as a brown solid (371.2 mg, 90% yield).

^{1}H NMR (400 MHz, $CDCl_3$): δ 8.47 (d, *J* = 9.2 Hz, 1H), 8.14–8.10 (m, 3H), 8.07 (d, *J* = 9.2 Hz, 1H), 8.00–7.96 (m, 2H), 7.92 (d, *J* = 8.9 Hz, 1H), 7.68 (d, *J* = 8.4 Hz, 1H), 5.07 (d, *J* = 2.4 Hz, 2H), 2.58 (t, *J* = 2.4 Hz, 1H). ESI mass: *m/z* calcd for $C_{19}H_{14}O$ $[M+K]^+$, 297.1; found: 296.8.

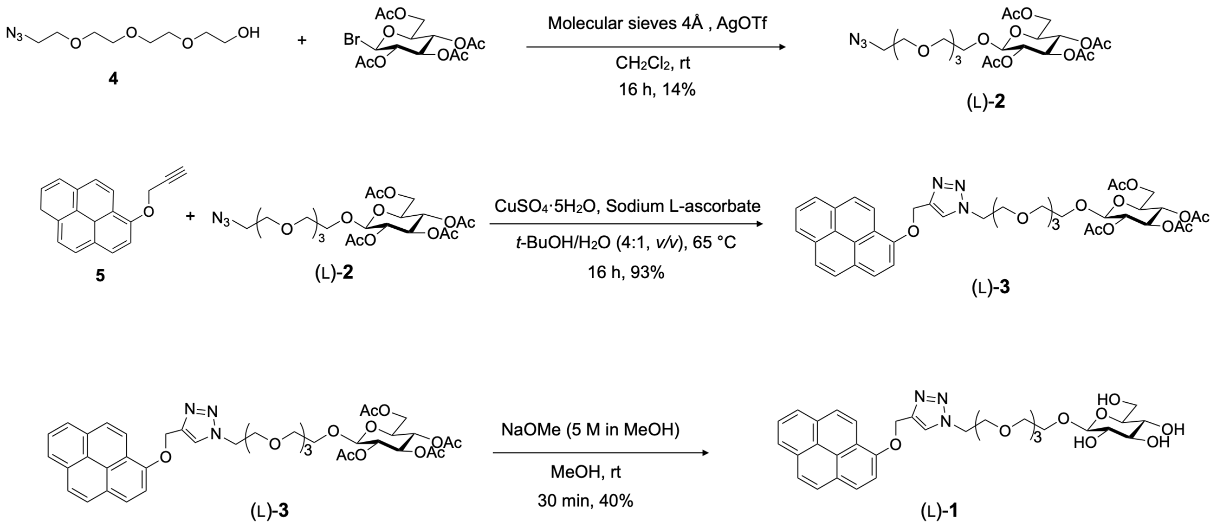

Scheme S2. Synthetic route to glucose-based chiral amphiphiles (L)-**1**.

Synthesis of (D)-2

Acetylated glycosyl bromide (300 mg, 0.73 mmol) was dissolved in DCM, followed by the addition of molecule **4** (223.9 mg, 1.02 mmol), molecular sieves 4 Å, and silver trifluoromethanesulfonate (AgOTf). The reaction mixture was stirred at room temperature for 16 h, and the reaction progress was monitored by TLC using CAM staining. After completion, the mixture was extracted with DCM, and the organic phase was dried over anhydrous $MgSO_4$. The filtrate was concentrated under reduced pressure, and the crude product was purified by silica gel flash column chromatography (ethyl acetate: n-hexane 2:1, *v/v*). The desired compound was obtained as a light-yellow oil (108.25 mg, 27% yield).

^{1}H NMR (400 MHz, $CDCl_3$): δ 5.19 (t, *J* = 9.5 Hz, 1H), 5.07 (t, *J* = 9.7 Hz, 1H), 5.02–4.95 (m, 1H), 4.60 (d, *J* = 8.0 Hz, 1H), 4.27–4.20 (m, 1H), 4.15–4.09 (m, 2H), 3.93 (dt, *J* = 11.1, 4.3 Hz, 1H), 3.76–3.60 (m, 13H), 3.38 (t, *J* = 5.1 Hz, 2H). ESI mass: *m/z* calcd for $C_{22}H_{35}N_3O_{13}$ $[M+Na]^+$, 572.2, $[M+K]^+$, 588.2; found: $[M+Na]^+$, 572.3, $[M+K]^+$, 588.3.

Synthesis of (L)-2

Acetylated glycosyl bromide (200 mg, 0.5 mmol) was dissolved in DCM, and molecule **4** (149.3 mg, 0.68 mmol), molecular sieves 4 Å, and silver trifluoromethanesulfonate (AgOTf) were added. The reaction mixture was stirred at room temperature for 16 h, and the reaction progress was monitored by TLC using CAM staining. The mixture was then extracted with DCM, and the organic phase was dried over anhydrous $MgSO_4$. The filtrate was concentrated under reduced pressure, and the crude product was purified by silica gel flash column chromatography (ethyl acetate: n-hexane 2:1, *v/v*). The desired compound was obtained as a light-yellow oil (38 mg, 14% yield).

^{1}H NMR (400 MHz, $CDCl_3$): δ 5.19 (t, *J* = 9.5 Hz, 1H), 5.08 (t, *J* = 9.7 Hz, 1H), 5.01–4.95 (m, 1H), 4.60 (d, *J* = 8.2 Hz, 1H), 4.27–4.20 (m, 1H), 4.15–4.09 (m, 2H), 3.93 (dt, *J* = 11.0, 4.2 Hz, 1H), 3.77–3.57 (m, 13H), 3.38 (t, *J* = 4.9 Hz, 2H). ESI mass: *m/z* calcd for $C_{22}H_{35}N_3O_{13}$ $[M+Na]^+$, 572.2; found: 572.3.

Synthesis of (D)-3

A mixture of (D)-**2** (80 mg, 0.15 mmol) and molecule **5** (100 mg, 0.39 mmol) was dissolved in *t*-BuOH/H_2O (4:1, *v/v*) containing catalytic amounts of $CuSO_4 \cdot 5H_2O$ (10 mol%) and sodium L-ascorbate (15 mol%). The mixture was stirred at 65 °C for 16 h under a nitrogen atmosphere, and the reaction progress was monitored by TLC. After cooling to room temperature, the reaction mixture was extracted with dichloromethane, and the organic phase was dried over anhydrous $MgSO_4$. The filtrate was concentrated under reduced pressure, and the crude product was purified by silica gel flash column chromatography (eluted with ethyl acetate) to afford the desired compound as a yellow oil (104 mg, 89% yield).

^{1}H NMR (400 MHz, $CDCl_3$): δ 8.44 (d, *J* = 9.2 Hz, 1H), 8.11 (dd, *J* = 8.0, 4.5 Hz, 3H), 8.03 (d, *J* = 9.1 Hz, 1H), 7.98–7.89 (m, 3H), 7.73 (d, *J* = 8.5 Hz, 1H), 5.60 (s, 2H), 5.12 (t, *J* = 9.4 Hz, 1H), 5.03 (t, *J* = 9.6 Hz, 1H), 4.95–4.91 (m, 1H), 4.58 (t, *J* = 5.0 Hz, 2H), 4.40 (d, *J* = 8.0 Hz, 1H), 4.18 (dd, *J* = 12.3, 4.6 Hz, 1H), 4.05 (dd, *J* = 12.4, 2.5 Hz, 1H), 3.87 (t, *J* = 5.0 Hz, 2H), 3.80 (dt, *J* = 10.9, 4.3 Hz, 1H), 3.68–3.43 (m, 12H), 2.04–1.98 (m, 12H). ESI mass: *m/z* calcd for $C_{41}H_{47}N_3O_{14}$ $[M+Na]^+$, 828.3, $[M+K]^+$, 844.3; found: $[M+Na]^+$, 828.6, $[M+K]^+$, 844.6.

Synthesis of (L)-3

A mixture of (L)-**2** (38 mg, 0.07 mmol) and molecule **5** (22.4 mg, 0.09 mmol) was dissolved in *t*-BuOH/H_2O (4:1, *v/v*) containing catalytic amounts of $CuSO_4 \cdot 5H_2O$ (10 mol%) and sodium L-ascorbate (15 mol%). The mixture was stirred at 65 °C for 16 h under a nitrogen atmosphere, and the reaction progress was monitored by TLC. After cooling to room temperature, the reaction mixture was extracted with dichloromethane, and the organic phase was dried over anhydrous $MgSO_4$. The filtrate was concentrated under reduced pressure, and the crude product was purified by silica gel flash column chromatography (eluted with ethyl acetate) to afford the desired compound as a yellow oil (52 mg, 93% yield).

^{1}H NMR (400 MHz, $CDCl_3$): δ 8.43 (d, *J* = 9.2 Hz, 1H), 8.10 (dd, *J* = 7.7, 4.3 Hz, 3H), 8.03 (d, *J* = 9.2 Hz, 1H), 7.96–7.89 (m, 3H), 7.72 (d, *J* = 8.4 Hz, 1H), 5.60 (s, 2H), 5.12 (t, *J* = 9.5 Hz, 1H), 5.03 (t, *J* = 9.6 Hz, 1H), 4.95–4.90 (m, 1H), 4.59–4.55 (m, 2H), 4.40 (d, *J* = 7.9 Hz, 1H), 4.18 (dd, *J* = 12.3, 4.6 Hz, 1H), 4.05 (dd, *J* = 12.3, 2.3 Hz, 1H), 3.87 (t, *J* = 5.0 Hz, 2H), 3.80 (dt, *J* = 10.8, 4.3 Hz, 1H), 3.60–3.42 (m, 12H), 2.04–1.98 (m, 12H). ESI mass: *m/z* calcd for $C_{41}H_{47}N_3O_{14}$ $[M+H]^+$, 806.3, $[M+Na]^+$, 828.3, $[M+K]^+$, 844.3; found: $[M+H]^+$, 806.6, $[M+Na]^+$, 828.6, $[M+K]^+$, 844.6.

Synthesis of (D)-1

(D)-**3** (104 mg, 0.13 mmol) was dissolved in methanol and 5 M sodium methoxide (MeONa) in methanol was added. The mixture was stirred for 30 min, and the reaction progress was monitored by TLC. Upon completion, the mixture was neutralized to pH 6 with ion-exchange resin. The reaction mixture was filtered, concentrated under reduced pressure, and the crude product was purified by preparative high-performance liquid chromatography (HPLC, C18 column, CH_3CN/H_2O = 40:60, *v/v*) to afford the desired compound as a light-yellow oil (38 mg, 46% yield). $[\alpha]_D^{25} = -6.7$ (c = 6.5 × 10^{-3} g mL^{-1}, MeOH).

^{1}H NMR (400 MHz, DMSO-d_6): δ 8.34 (d, *J* = 4.5 Hz, 1H), 8.31–8.26 (m, 1H), 8.24–8.15 (m, 2H), 8.12–8.07 (m, 2H), 8.03 (d, *J* = 7.6 Hz, 1H), 8.00–7.97 (m, 2H), 5.57 (s, 2H), 4.95 (d, *J* = 5.0 Hz, 1H), 4.91 (d, *J* = 4.7 Hz, 1H), 4.87 (d, *J* = 6.0 Hz, 1H), 4.57 (t, *J* = 5.2 Hz, 2H), 4.47 (t, *J* = 5.9 Hz, 1H), 4.12 (d, *J* = 7.8 Hz, 1H), 3.85–3.80 (m, 2H), 3.65 (ddd, *J* = 12.1, 5.9, 1.8 Hz, 1H), 3.53–3.37 (m, 13H). ^{13}C NMR (100 MHz, DMSO-d_6): δ 151.93, 142.56, 131.09, 130.94, 127.17, 126.35, 126.31, 125.80, 124.96, 124.85, 124.82, 124.80, 124.38, 124.16, 123.98, 120.76, 119.49, 110.41, 102.87, 76.78, 76.65, 73.27, 69.93, 69.69, 69.61, 69.56, 69.53, 69.48, 69.43, 68.58, 67.66, 62.31, 60.96, 49.39. ESI mass: *m/z* calcd for $C_{33}H_{39}N_3O_{10}$ $[M+H]^+$, 638.3, $[M+Na]^+$, 660.3, $[M+K]^+$, 676.3; found: $[M+H]^+$, 638.2, $[M+Na]^+$, 660.2, $[M+K]^+$, 676.2.

Synthesis of (L)-1

(L)-**3** (54 mg, 0.07 mmol) was dissolved in methanol and 5 M MeONa in methanol was added. The mixture was stirred for 30 min, and the reaction progress was monitored by TLC. Upon completion, the mixture was neutralized to pH 6 with ion-exchange resin. The reaction mixture was filtered, concentrated under reduced pressure, and the crude product was purified by preparative HPLC (C18 column, CH_3CN/H_2O = 40:60, *v/v*) to afford the desired compound as a light-yellow oil (17 mg, 40% yield). $[\alpha]_D^{25} = +6.9$ (c = 6.5 × 10^{-3} g mL^{-1}, MeOH).

^{1}H NMR (400 MHz, DMSO-d_6): δ 8.35 (d, *J* = 4.9 Hz, 1H), 8.33–8.27 (m, 1H), 8.23–8.19 (m, 2H), 8.14–8.07 (m, 2H), 8.04 (d, *J* = 7.7 Hz, 1H), 8.01–7.98 (m, 2H) 5.57 (s, 2H), 4.95 (d, *J* = 5.0 Hz, 1H), 4.91 (d, *J* = 4.8 Hz, 1H), 4.88 (d, *J* = 4.8 Hz, 1H), 4.58 (t, *J* = 5.2 Hz, 2H), 4.48 (t, *J* = 5.9 Hz, 1H), 4.12 (d, *J* = 7.8 Hz, 1H), 3.87–3.82 (m, 2H), 3.65 (ddd, *J* = 11.8, 5.9, 1.8 Hz, 1H), 3.58–3.34 (m, 13H). ^{13}C NMR (100 MHz, DMSO-d_6): δ 152.57, 143.20, 131.73, 131.58, 127.82, 127.04, 127.00, 126.96, 126.45, 125.60, 125.49, 125.47, 125.03, 124.81, 124.63, 121.48, 121.41, 111.07, 103.50, 77.41, 77.29, 73.91, 70.57, 70.52, 70.34, 70.25, 70.19, 70.12, 70.07, 69.22, 68.30, 62.95, 61.60, 50.03. ESI mass: *m/z* calcd for $C_{33}H_{39}N_3O_{10}$ $[M+Na]^+$, 660.3, $[M+K]^+$, 676.3; found: $[M+Na]^+$, 660.2, $[M+K]^+$, 676.2.

Section 2. Supplementary figures

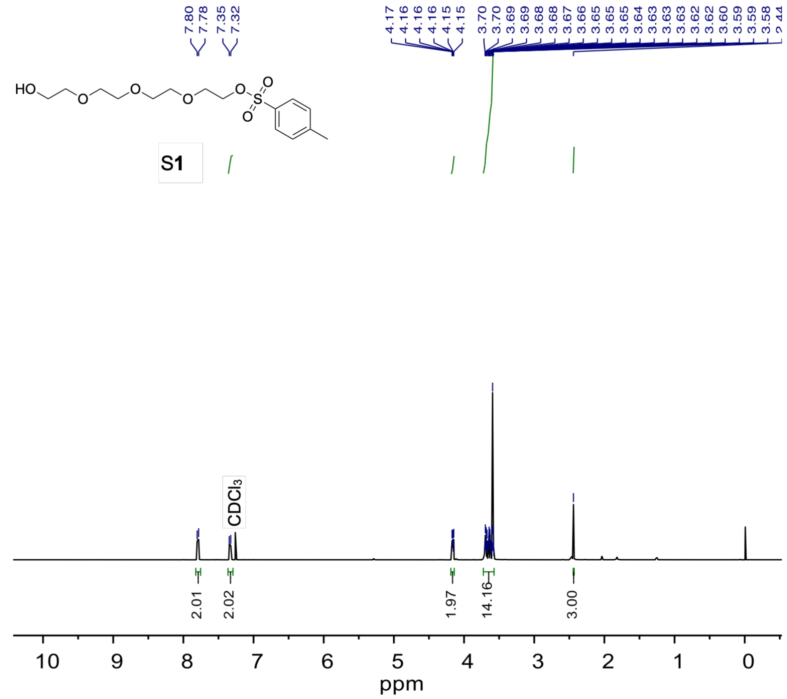

Figure S1. ^{1}H-NMR spectrum of molecule S**1** in $CDCl_3$.

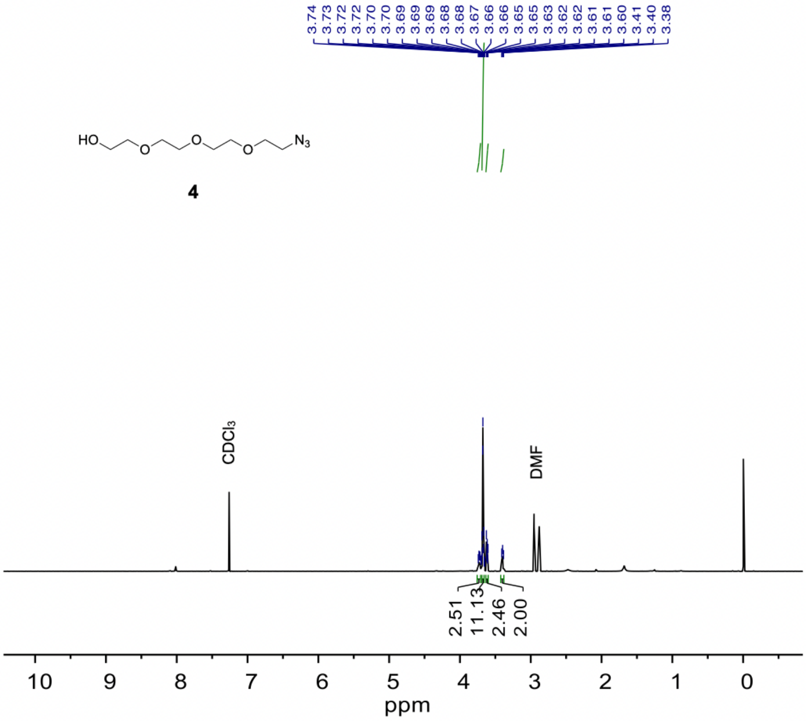

Figure S2. ^{1}H-NMR spectrum of molecule **4** in $CDCl_3$.

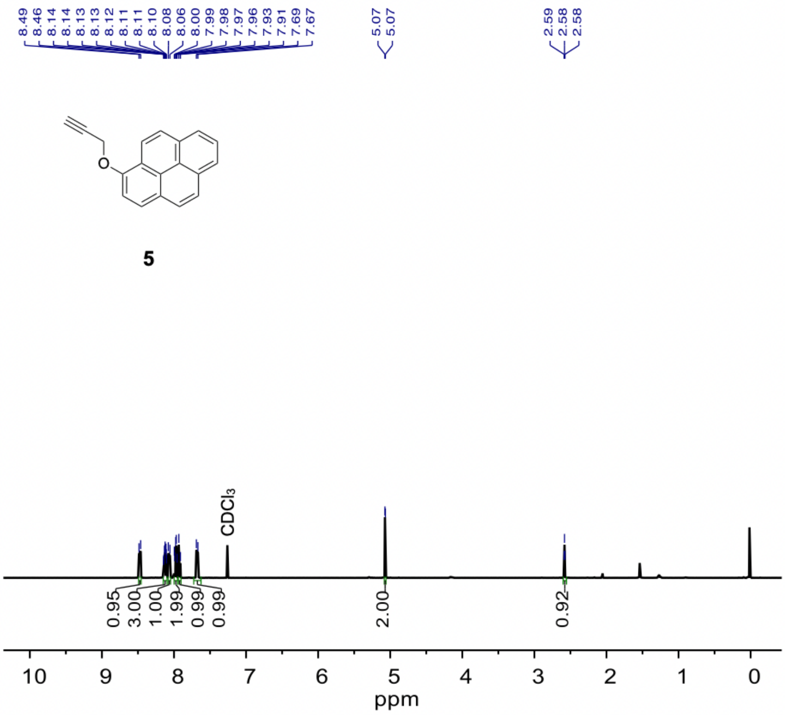

Figure S3. ^{1}H-NMR spectrum of molecule **5** in $CDCl_3$.

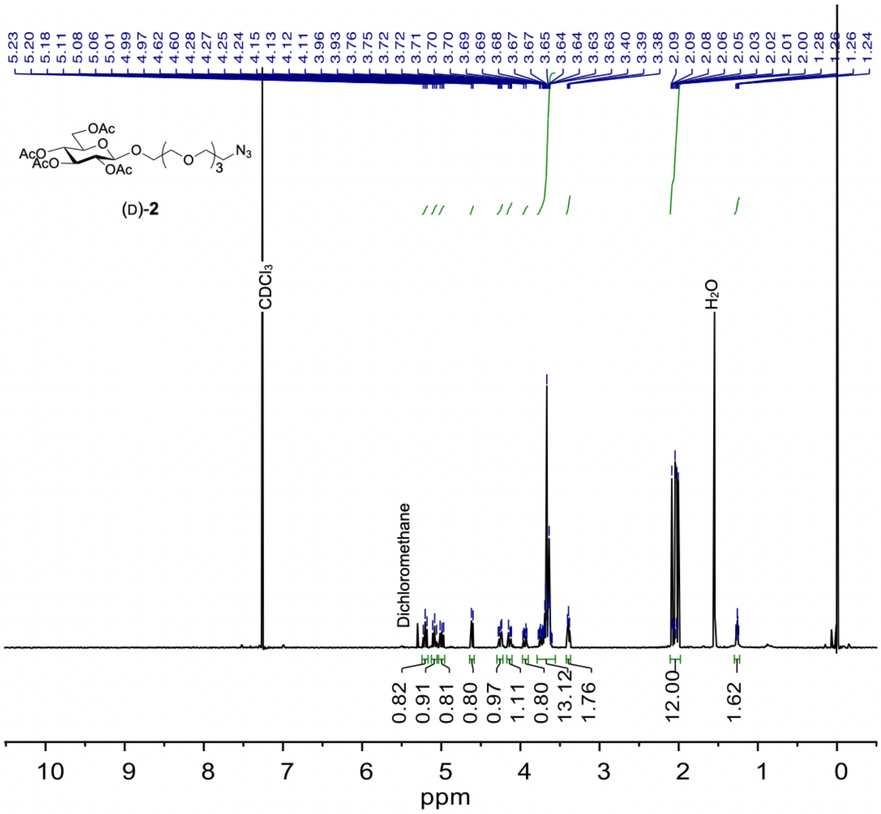

Figure S4. ^{1}H-NMR spectrum of (D)-**2** in $CDCl_3$.

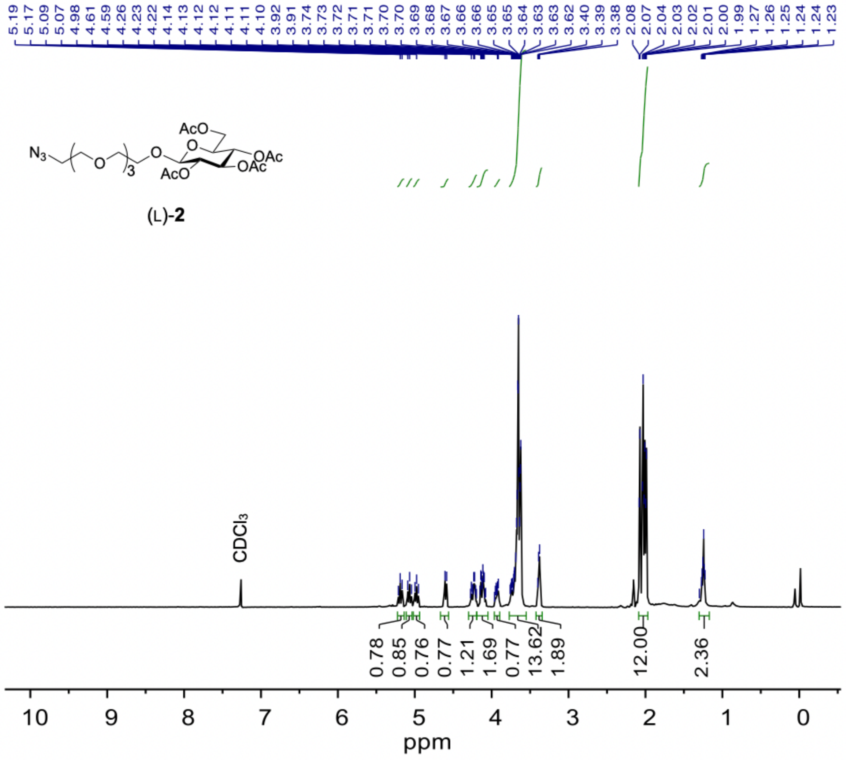

Figure S5. ^{1}H-NMR spectrum of (L)-**2** in $CDCl_3$.

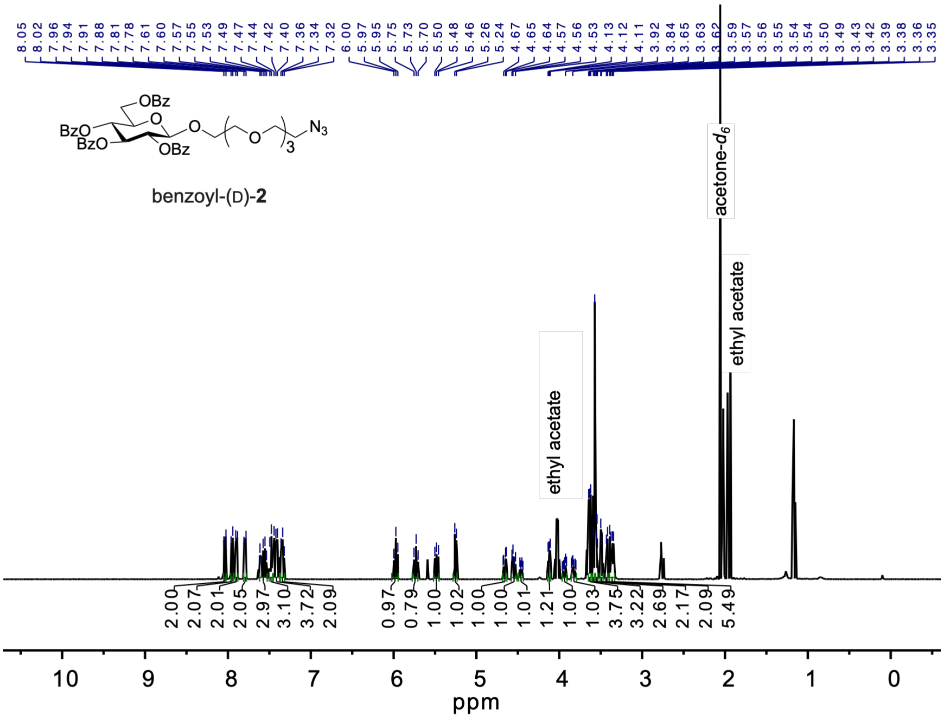

Figure S6. ^{1}H NMR spectrum of benzoyl-(D)-**2** in acetone-d_6.

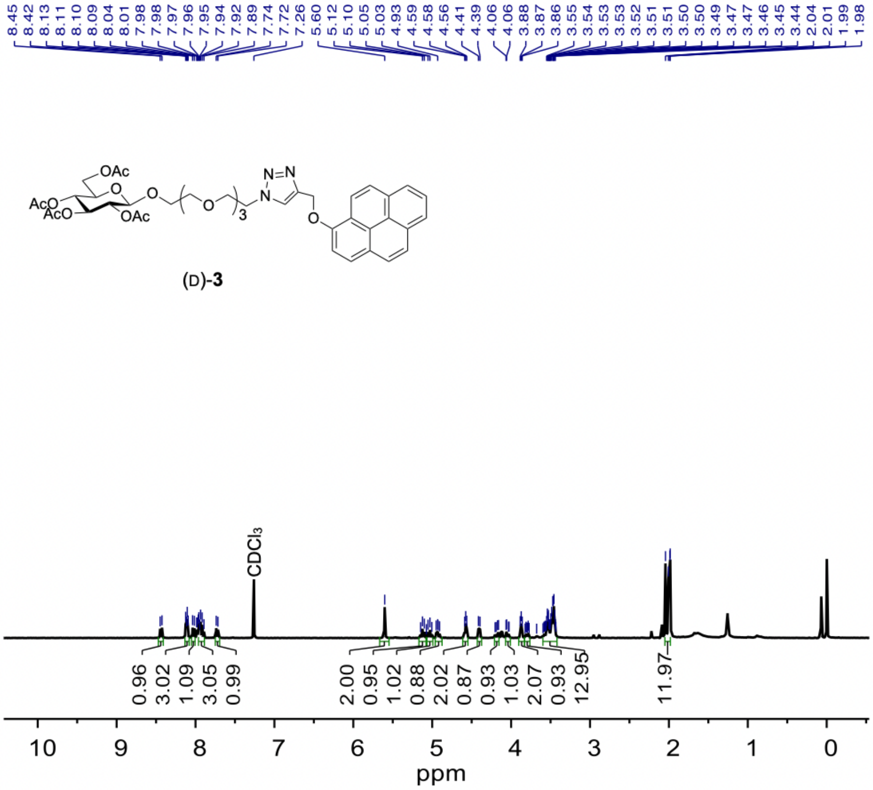

Figure S7. ^{1}H-NMR spectrum of (D)-**3** in $CDCl_3$.

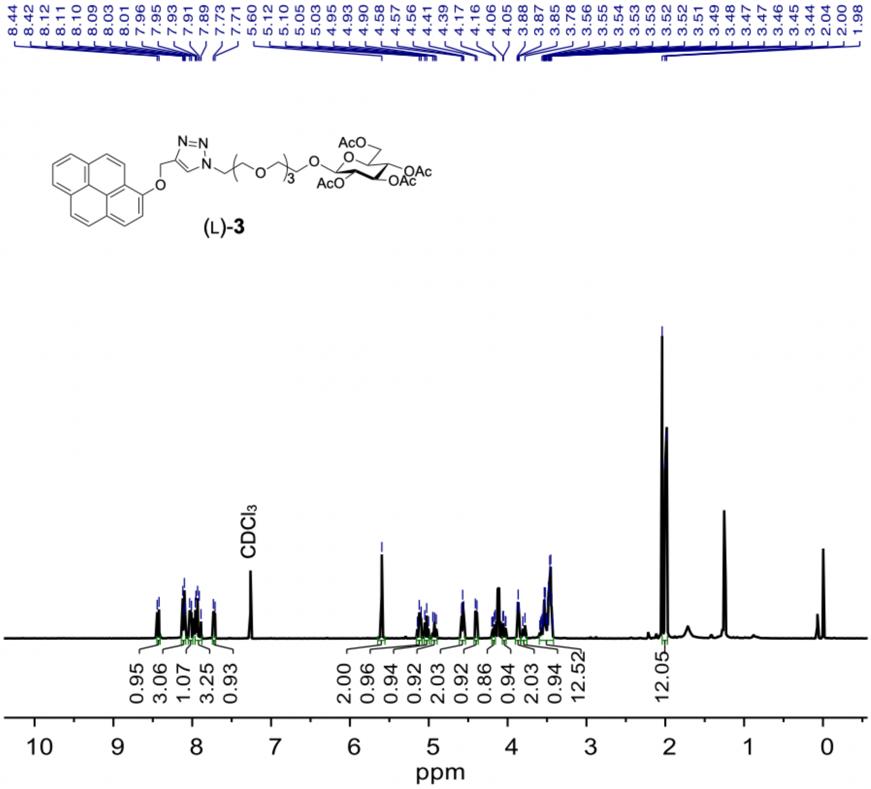

Figure S8. ^{1}H-NMR spectrum of (L)-**3** in $CDCl_3$.

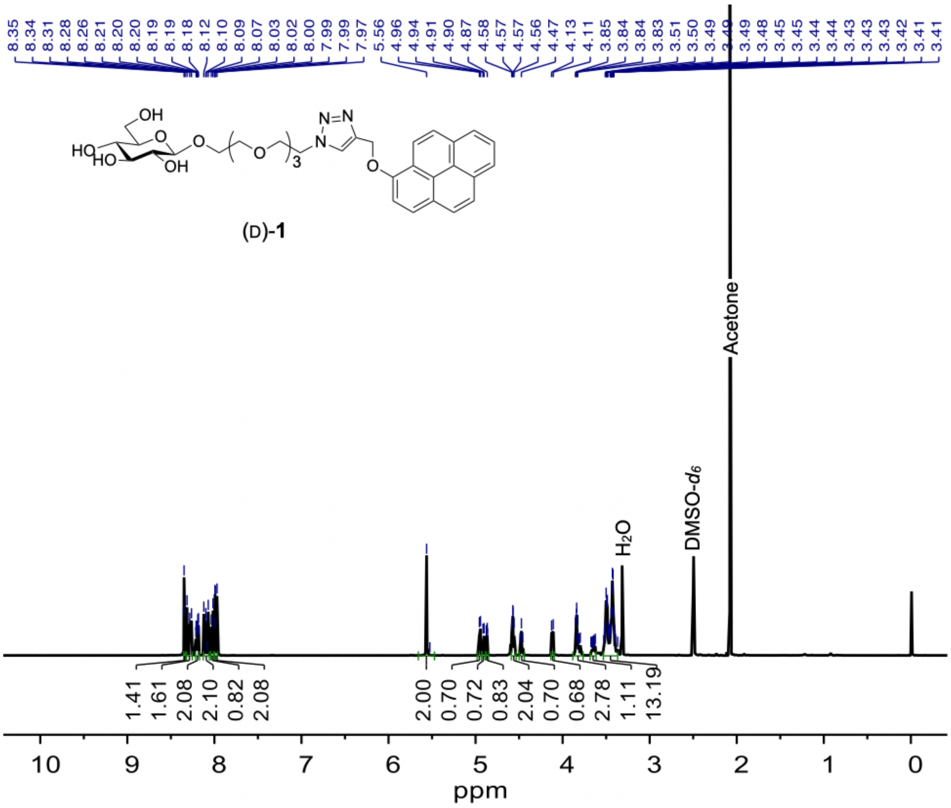

Figure S9. ^{1}H-NMR spectrum of (D)-**1** in DMSO-d_6.

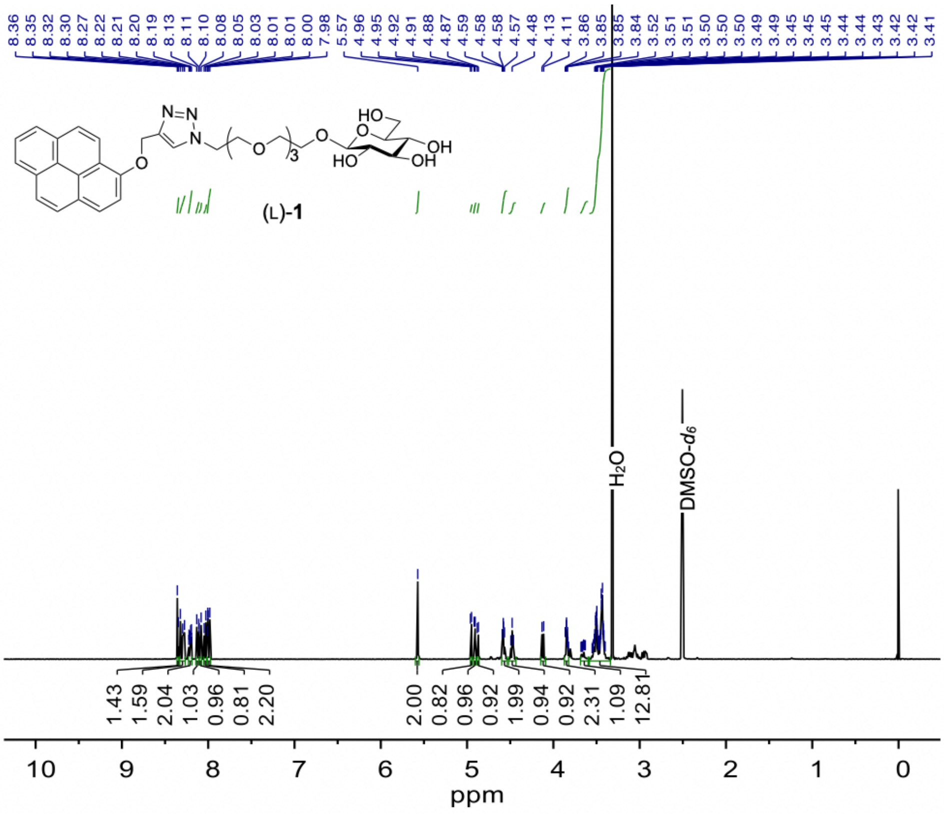

Figure S10. ^{1}H-NMR spectrum of (L)-**1** in DMSO-d_6.

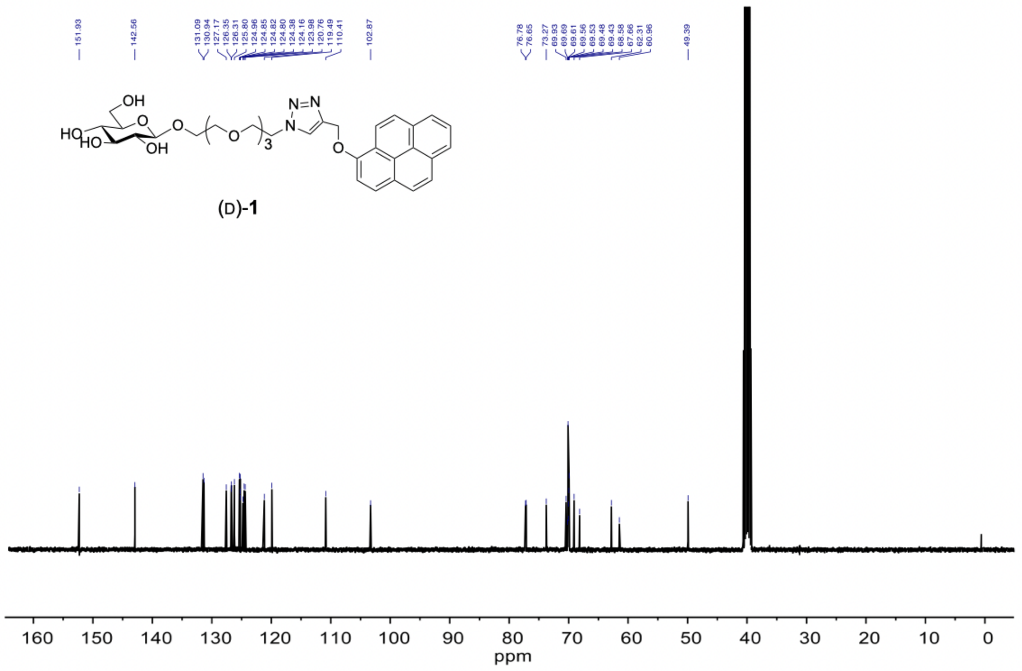

Figure S11. ^{13}C-NMR spectrum of (D)-**1** in DMSO-d_6.

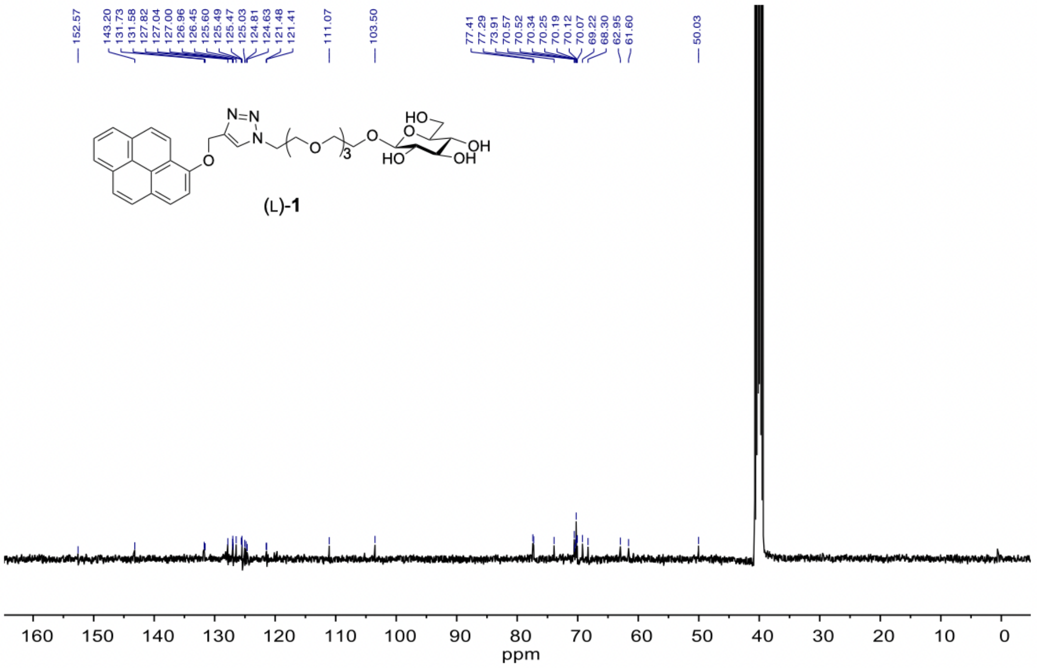

Figure S12. ^{13}C-NMR spectrum of (L)-**1** in DMSO-d_6.

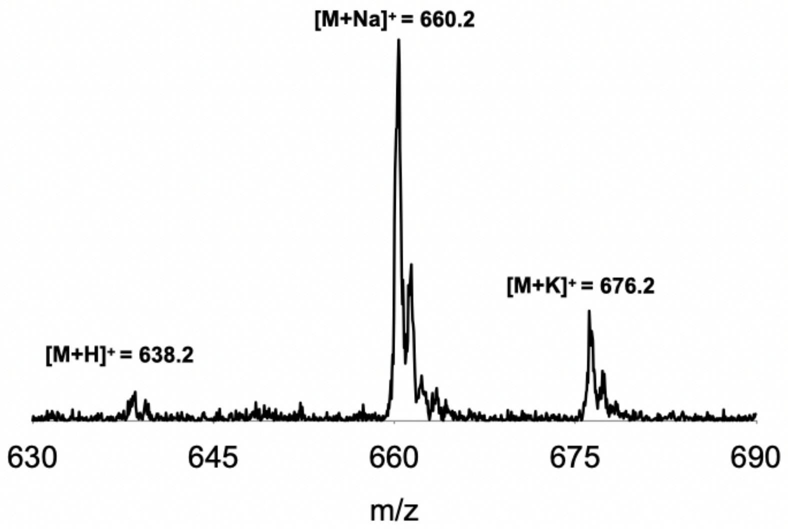

Figure S13. ESI-mass spectrum of (D)-**1**.

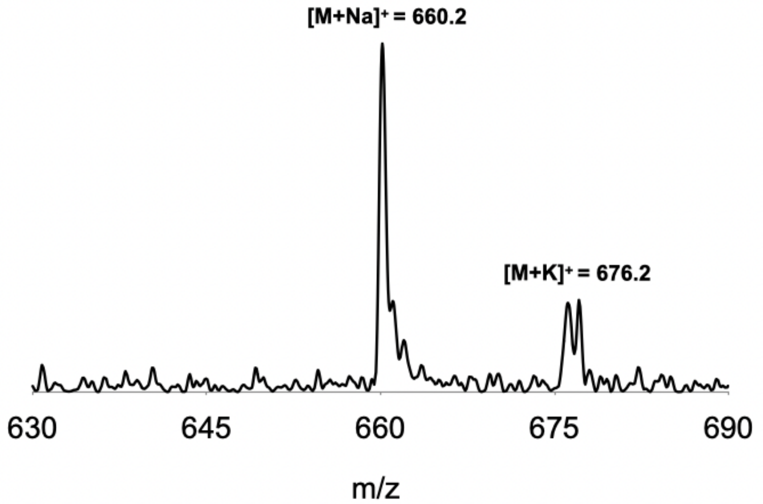

Figure S14. ESI-mass spectrum of (L)-**1**.

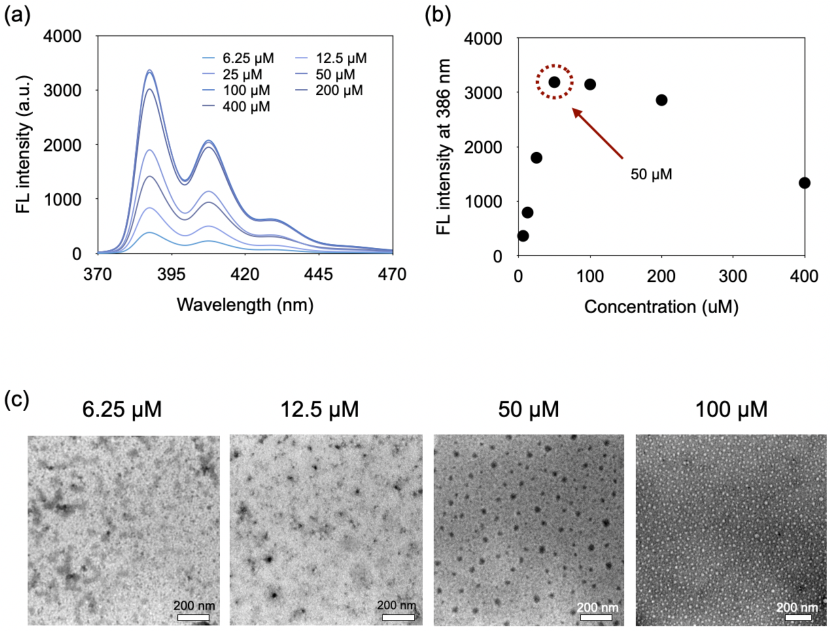

Figure S15. (a) Emission spectra of (D)-**1** at different concentrations in aqueous solution. Excitation wavelength: 342 nm. (b) The fluorescence emission intensity at 386 nm at different concentrations. (c) Transmission electron microscopy (TEM) images obtained at 6.25, 12.5, 50, and 100 µM, indicating the formation of particles above 50 µM

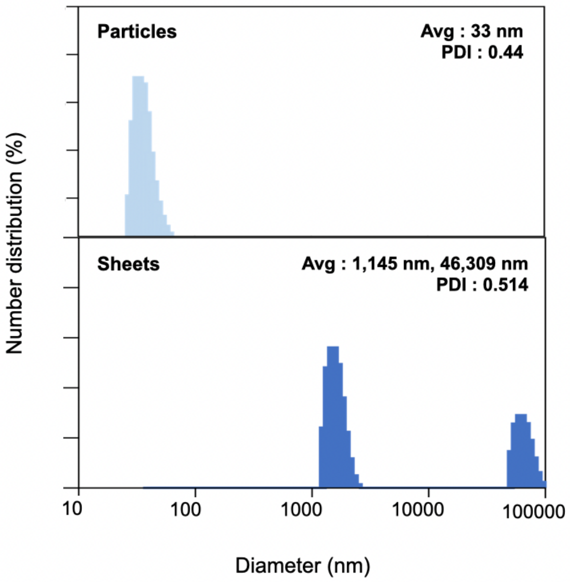

Figure S16. Dynamic light scattering (DLS) analysis of self-assembled amphiphilic (D)-**1** (314 μM) and its co-assembly with 1.0 equivalent of octafluoronaphthalene (OFN) in aqueous solution. Self-assembled (D)-**1** formed small aggregates (particles) with an average diameter of approximately 33 nm, and the addition of OFN resulted in the formation of larger aggregates (sheets) with diameters of several micrometers. This demonstrates that the addition of OFN induces the formation of large-scale structures.

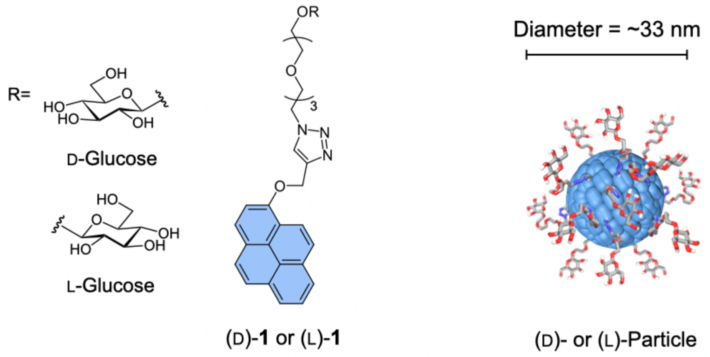

	Volume of (D)-1 or (L)-1 (Å³)	Volume of (D)-1 or (L)-1 (nm³)	Radius of particle	Volume of particle (nm³)	Number of (D)-1 or (L)-1 units	Number of (D)-1 or (L)-1 units per one particle
**VDW***	537.381	0.537381	16.5 nm	(4/3) x π x 16.5^3 = 18807.03	18807.03 / 0.537381 = 34998	34998
**Solvent****	572.849	0.572849	16.5 nm	(4/3) x π x 16.5^3 = 18807.03	18807.03 / 0.572849 =32831	32831

*VDW: Creates a surface based on the van der Waals (VDW) radius of each atom in molecule.
**Solvent: Creates a solvent surface, similar to a Connolly solvent surface.

Figure S17. Calculation of the number of (D)-**1** or (L)-**1** units per one particle. The particle volume was calculated to be 18807 nm³, and the molecular volume of (D)-**1** or (L)-**1** was estimated to be 537.4 Å³ (van der Waals) and 572.8 Å³ (solvent-accessible), respectively. Based on these values, each particle is expected to contain 32831–34998 molecules of (D)-**1** or (L)-**1**.

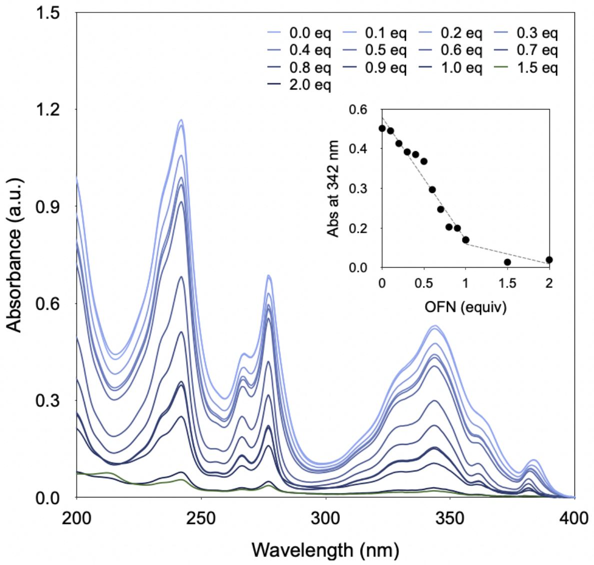

Figure S18. UV–vis absorption spectra of (D)-**1** (314 μM) upon addition of OFN. The absorbance at 342 nm gradually decreased upon the addition of OFN, indicating enhanced π–π interactions between the pyrene moiety of (D)-**1** and OFN.

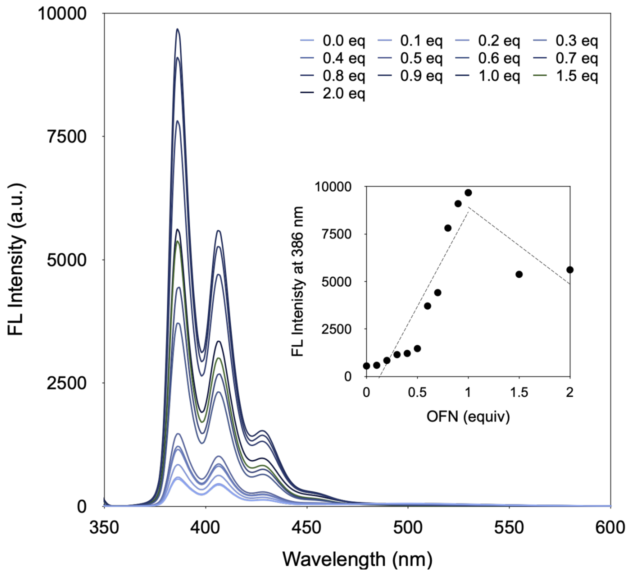

Figure S19. Emission spectra of (D)-**1** (314 μM) upon addition of OFN (λ_{ex} = 342 nm). The fluorescence emission intensity at 386 nm gradually increased and reached a maximum upon the addition of 1 equivalent of OFN. This indicates strong intermolecular π–π stacking between the pyrene moiety of (D)-**1** and OFN.

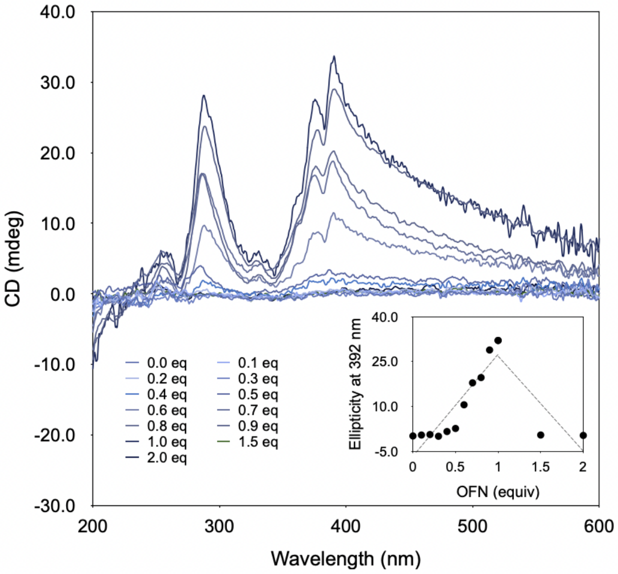

Figure S20. Circular dichroism (CD) spectra of (D)-**1** (314 μM) upon addition of OFN. The ellipticity at 392 nm increased and reached a maximum at 1 equivalent of OFN, indicating the formation of a chiral π–π stacked structure between the pyrene moiety of (D)-**1** and OFN.

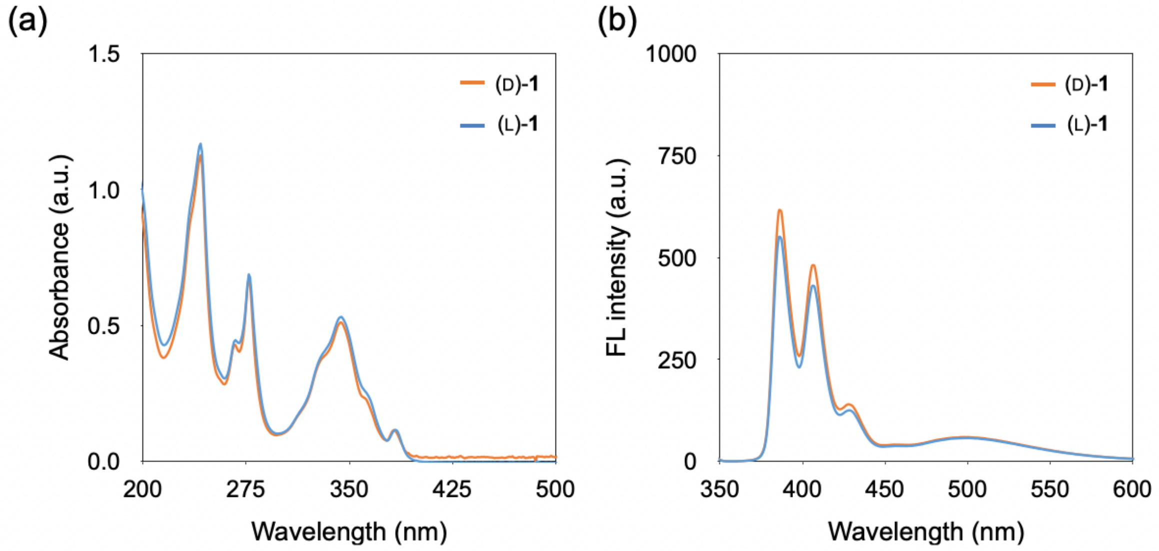

Figure S21. (a) Absorption and (b) emission spectra of (D)-**1** and (L)-**1** (314 μM) in aqueous solution (λ_{ex} = 342 nm). (D)-**1** and (L)-**1** exhibited identical absorption and emission spectra, indicating that they show the same spectral behavior in aqueous solution.

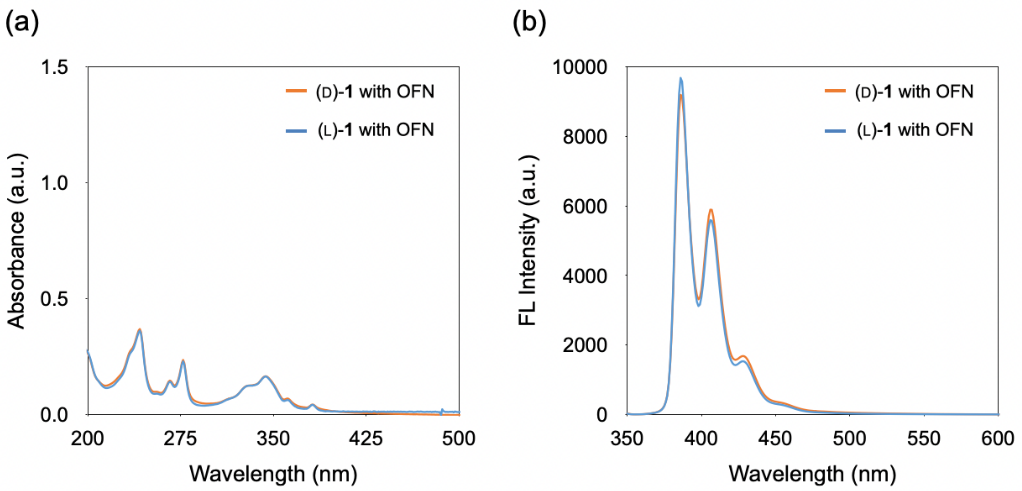

Figure S22. (a) Absorption and (b) emission spectra of (D)-**1** and (L)-**1** (314 μM) in aqueous solution containing OFN (λ_{ex} = 342 nm). Both (D)-**1** and (L)-**1** in the presence of OFN exhibited identical absorption and emission spectra, indicating that they show the same spectral behavior in aqueous solution.

.

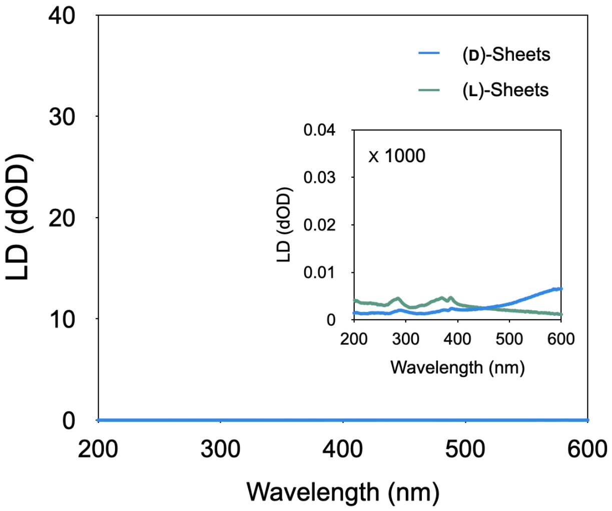

Figure S23. Linear dichroism (LD) spectra of (D)-sheets and (L)-sheets in aqueous solution. No distinct LD peaks appeared, and only extremely weak signals were visible when the Y-axis was magnified 1000-fold. This result confirms that the chiral signals observed in the CD spectrum are not an LD-related artifact, but instead originate from the inherent supramolecular chirality of the co-assembly.

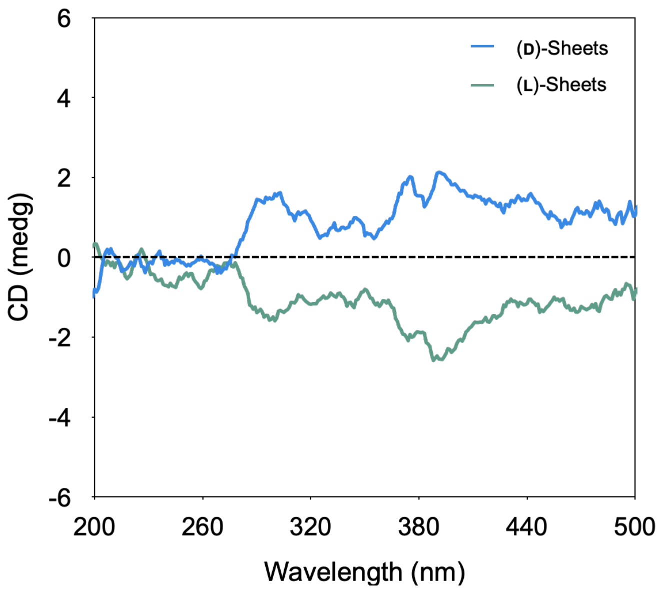

Figure S24. The CD spectra of (D)-sheets and (L)-sheets in the solid state. The sample was prepared as a film by drop-casting sheets formed from an aqueous solution onto a CD cuvette, followed by drying. The CD profiles exhibit mirror-image signals similar to those observed in solution.

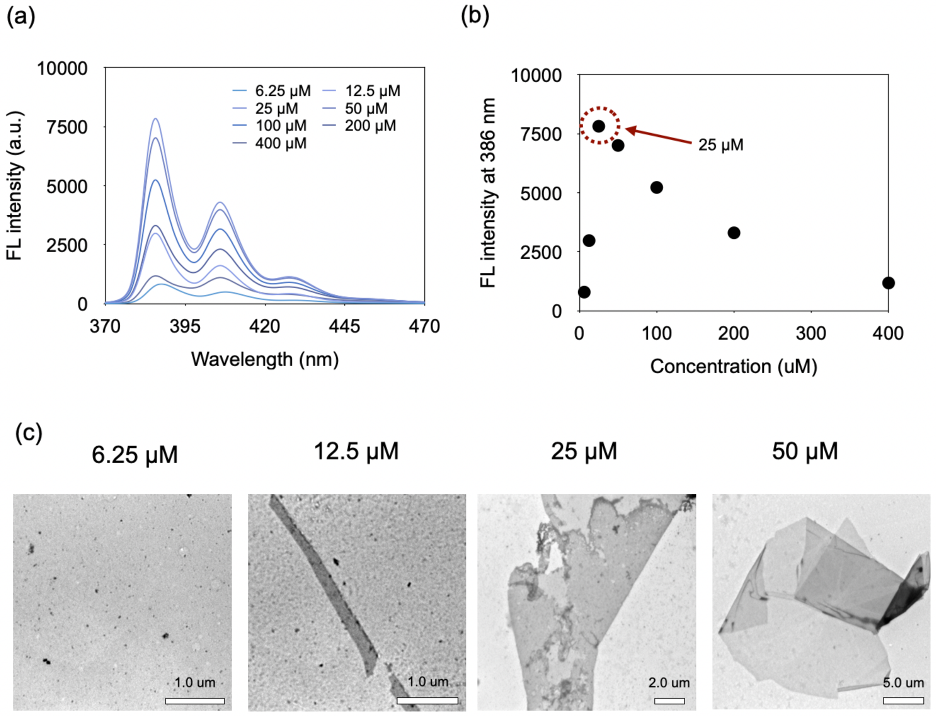

Figure S25. (a) Emission spectra of (D)-**1** with OFN at different concentrations in aqueous solution. Excitation wavelength: 342 nm. (b) The fluorescence emission intensity at 386 nm at different concentrations. (c) TEM images obtained at 6.25, 12.5, 25, and 50 µM, indicating the formation of a sheet above 25 µM.

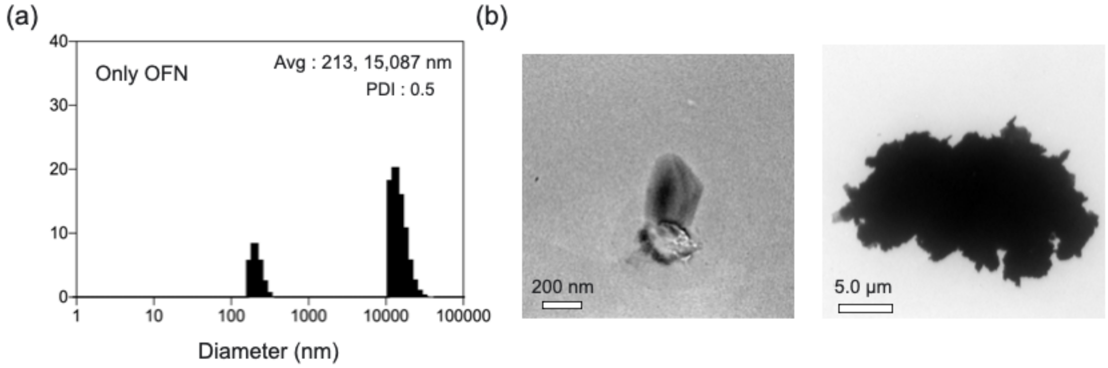

Figure S26. (a) DLS profile of OFN (314 μM) in aqueous solution. (b) TEM images of OFN aggregates showing irregular morphologies. These data indicate that OFN alone forms disordered bulk aggregates rather than thin, extended sheets, confirming that the sheet morphology arises specifically from the co-assembly of (D)-**1** and OFN.

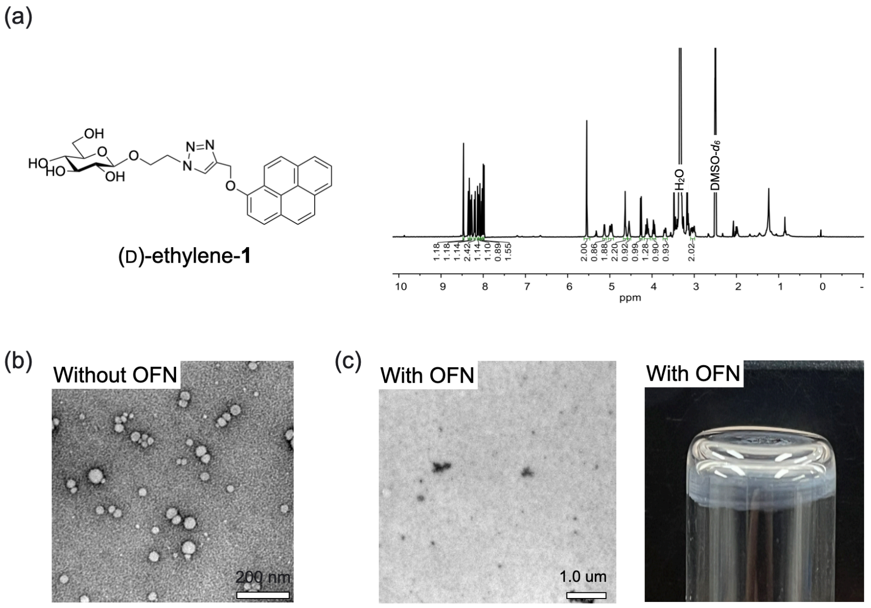

Figure S27. (a) Chemical structure of (D)-ethylene-**1** and its ¹H NMR spectrum in DMSO-d_6. (b) TEM image of (D)-ethylene-**1** in an aqueous solution (314 μM). (c) TEM image and vial image of (D)-ethylene-**1** with OFN in aqueous solution (314 μM). The mixture of (D)-ethylene-**1** and OFN showed poor solubility in aqueous solution and remained as an undispersed film. This result indicates that both adequate aqueous solubility and sufficient linker length are essential for sheet formation.

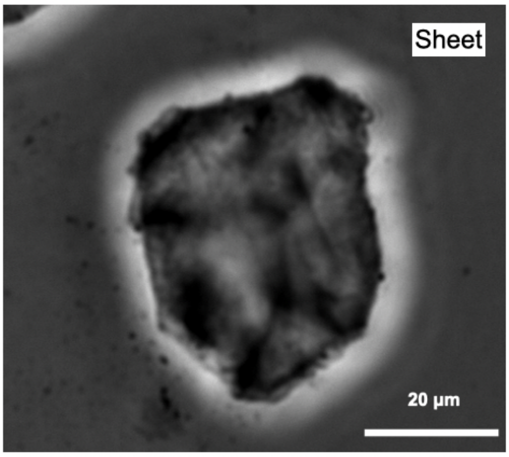

Figure S28. Phase-contrast optical microscopy (OM) image of a sheet formed upon addition 1 equivalent of OFN to (D)-**1** (314 μM) in aqueous solution.

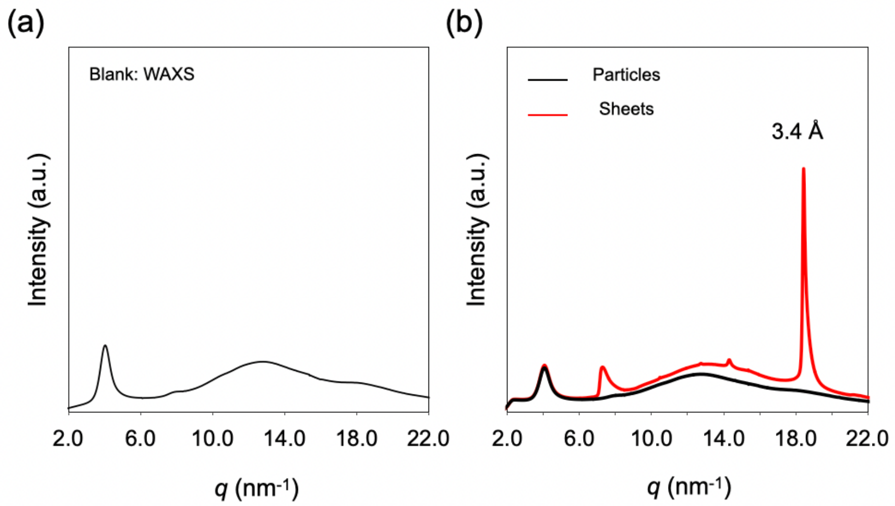

Figure S29. (a) Wide-angle X-ray scattering (WAXS) pattern of a blank sample. (b) WAXS pattern of particles and sheets after freeze-drying. The strong reflection at ~3.4 Å in the sheets corresponds to aligned π–π stacking with face-to-face, which was not observed in the particles.

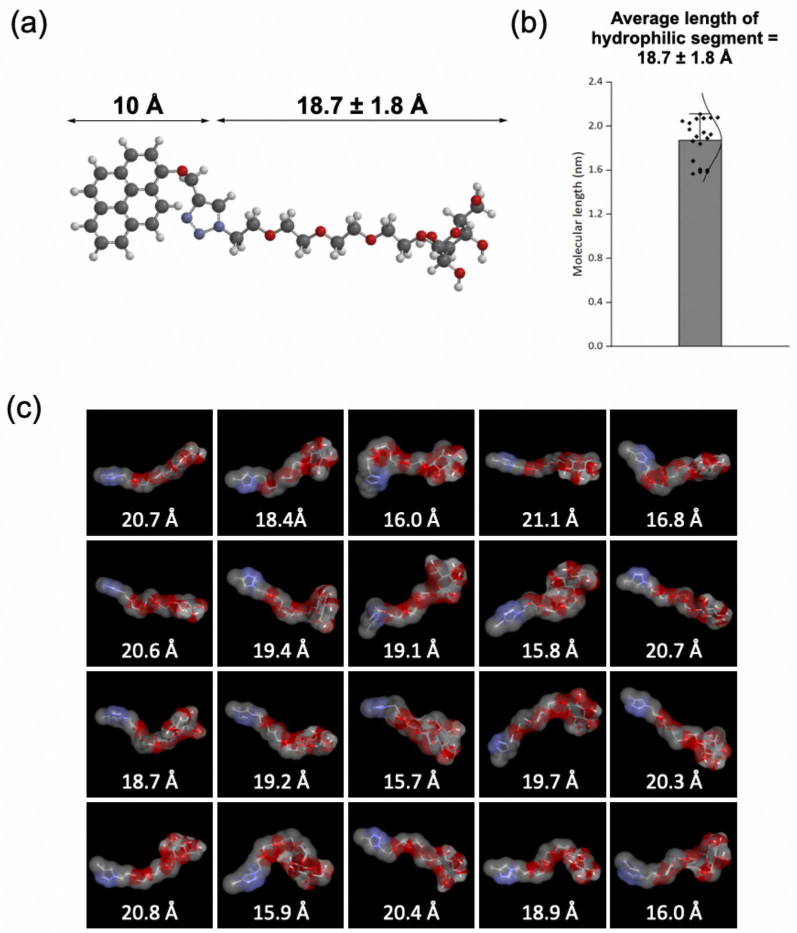

Figure S30. (a) Molecular dimensions of (D)-**1** from molecular dynamics (MD) simulations in aqueous solution. The hydrophobic pyrene unit was ~10 Å, and the average length of the flexible hydrophilic segment was 18.7 ± 1.8 Å. (b) The calculated average length of 20 representative hydrophilic glucose chains. (c) Representative snapshots of twenty hydrophilic segment conformers obtained during the MD simulation.

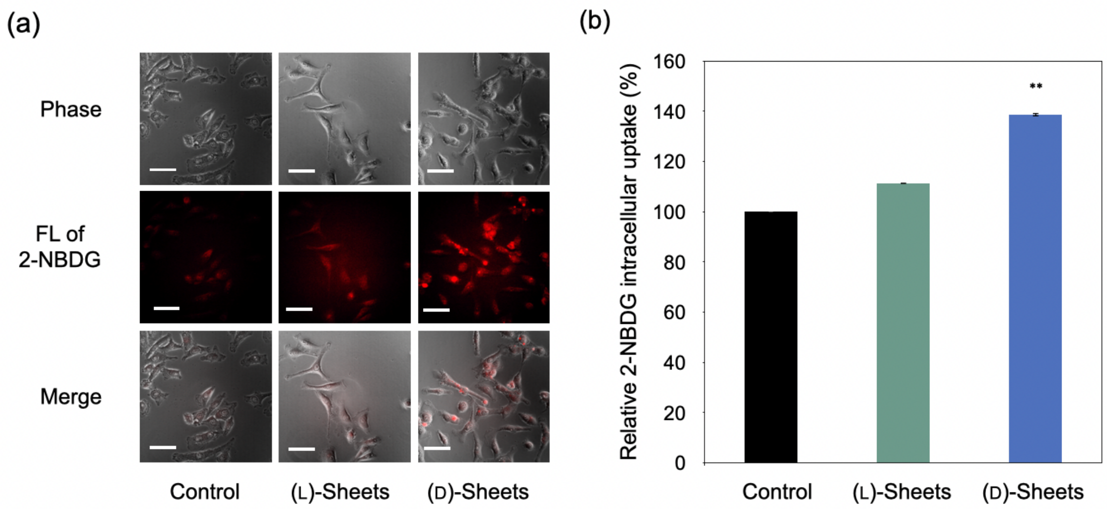

Figure S31. (a, b) Fluorescence optical microscopy (FOM) images and quantitative analysis of 2-deoxy-2-[(7-nitro-2,1,3-benzoxadiazol-4-yl)amino]-D-glucose (2-NBDG) uptake of cells treated with (L)- and (D)-sheets at 50 μM (scale bar = 50 μm). Cells treated with (D)-sheets showed approximately 35% higher 2-NBDG uptake than those treated with (L)-sheets, indicating that (D)-sheets promote 2-NBDG uptake via GLUT on the cell membrane. The data are represented as mean ± SD (n = 3). $^{**}p < 0.01$.

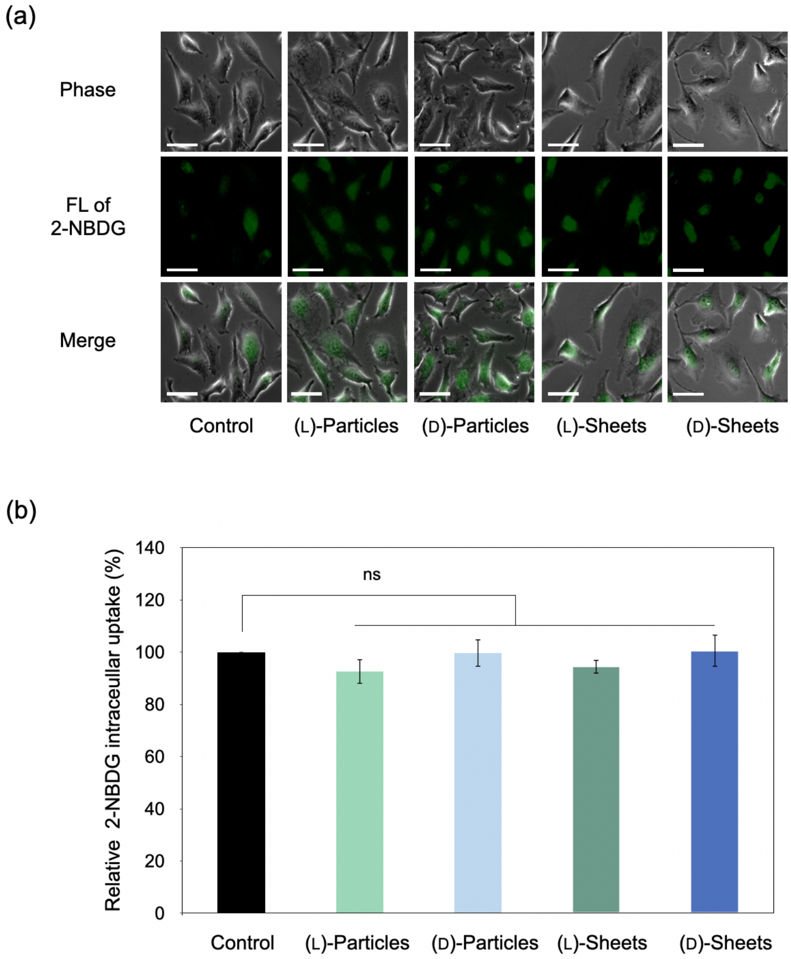

Figure S32. (a–b) Fluorescence optical microscopy (FOM) images and quantitative analysis of 2-NBDG uptake in HeLa cells treated with (L)- and (D)-particles and (L)-and (D)-sheets below critical aggregation concentration (6.25 μM). These results show that monomeric (D)-**1** or (L)-**1** does not influence GLUT1 activity, and that the supramolecular architecture formed at higher concentrations is required for the observed biological effect (scale bar = 50 μm).

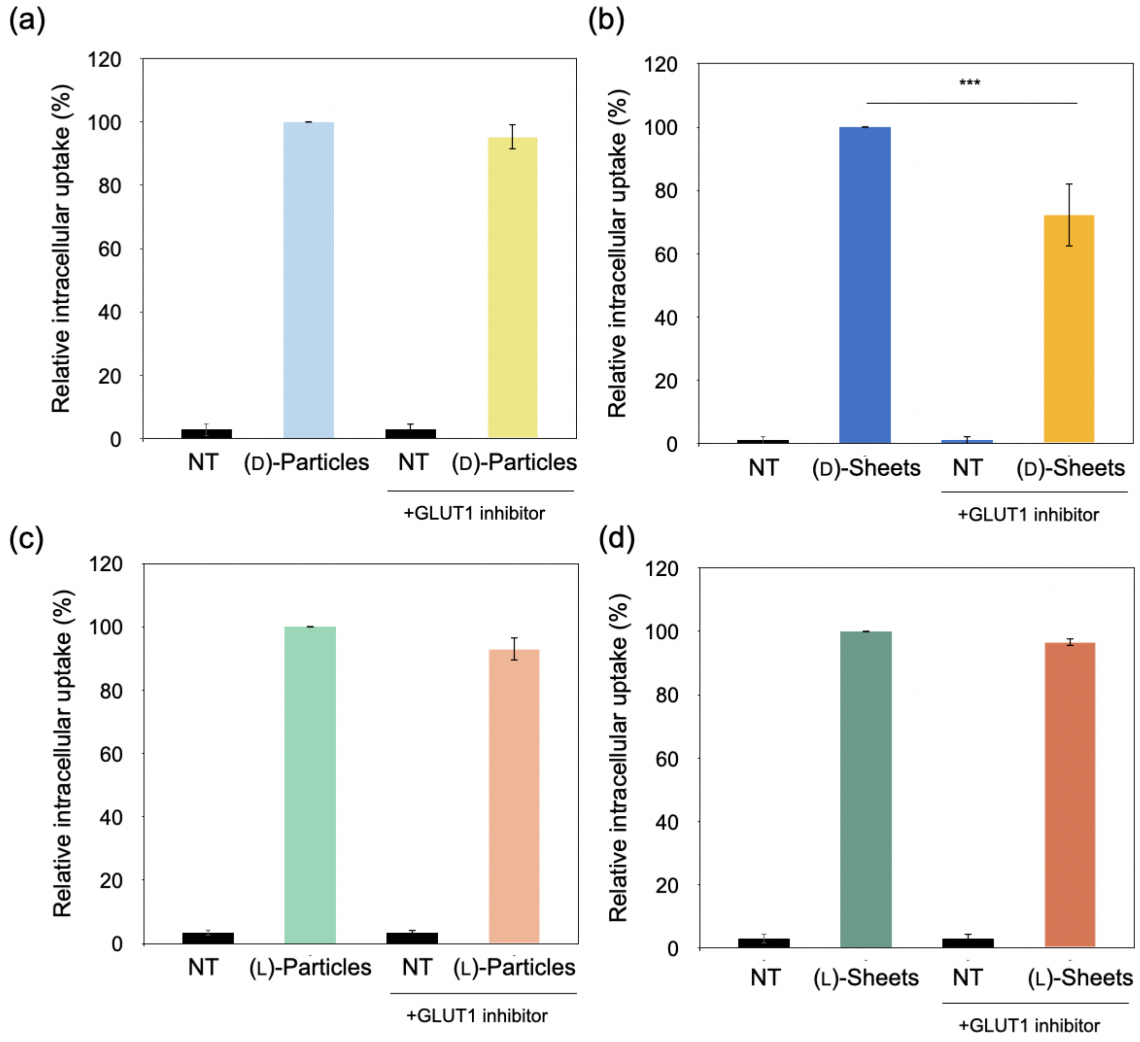

Figure S33. The relative cellular uptake of (a–d) (D)-particles, (D)-sheets, (L)-particles, and (L)-sheets was measured in the presence or absence of cytochalasin B, a GLUT1 inhibitor. In the presence of the GLUT1 inhibitor, uptake decreased by approximately 30% only for (D)-sheets, indicating a specific interaction with GLUT1. The data are represented as mean ± SD (n = 3). ***$p < 0.001$.
